# The SARS-CoV-2 monoclonal antibody combination, AZD7442, is protective in non-human primates and has an extended half-life in humans

**DOI:** 10.1126/scitranslmed.abl8124

**Published:** 2022-01-25

**Authors:** Yueh-Ming Loo, Patrick M. McTamney, Rosalinda H. Arends, Michael E. Abram, Anastasia A. Aksyuk, Seme Diallo, Daniel J. Flores, Elizabeth J. Kelly, Kuishu Ren, Richard Roque, Kim Rosenthal, Katie Streicher, Kevin M. Tuffy, Nicholas J. Bond, Owen Cornwell, Jerome Bouquet, Lily I. Cheng, James Dunyak, Yue Huang, Anton I. Rosenbaum, Venkatesh Pilla Reddy, Hanne Andersen, Robert H. Carnahan, James E. Crowe, Ana I. Kuehne, Andrew S. Herbert, John M. Dye, Helen Bright, Nicole L. Kallewaard, Menelas N. Pangalos, Mark T. Esser

**Affiliations:** ^1^ Virology and Vaccine Discovery, Vaccines and Immune Therapies, BioPharmaceuticals R&D, AstraZeneca, Gaithersburg, MD, 20878, USA.; ^2^ Clinical Pharmacology and Quantitative Pharmacology, Clinical Pharmacology and Safety Sciences, BioPharmaceuticals R&D, AstraZeneca, Gaithersburg, MD, 20878, USA.; ^3^ Translational Medicine, Vaccines and Immune Therapies, BioPharmaceuticals R&D, AstraZeneca, Gaithersburg, MD 20878, USA.; ^4^ Analytical Sciences, BioPharmaceuticals R&D, AstraZeneca, Granta Park, Cambridge, CB21 6GH, UK.; ^5^ Integrated Bioanalysis, Clinical Pharmacology and Quantitative Pharmacology, Clinical Pharmacology and Safety Sciences, R&D, AstraZeneca, San Francisco, CA, 94080, USA.; ^6^ Oncology Safety Pathology, Clinical Pharmacology and Safety Sciences, BioPharmaceuticals R&D, AstraZeneca, Gaithersburg, MD 20878, USA.; ^7^ Clinical Pharmacology and Pharmacometrics, BioPharmaceuticals R&D, AstraZeneca, Gaithersburg, MD 20878, USA.; ^8^ Clinical Pharmacology and Pharmacometrics, BioPharmaceuticals R&D, AstraZeneca, Granta Park, Cambridge, CB21 6GH, UK.; ^9^ BIOQUAL Inc., Rockville, MD 20850, USA.; ^10^ Department of Pediatrics, Vanderbilt University Medical Center, Nashville, TN 37232, USA.; ^11^ The Vanderbilt Vaccine Center, Vanderbilt University Medical Center, Nashville, TN, 37232, USA.; ^12^ Department of Pathology, Microbiology and Immunology, Vanderbilt University, Nashville, TN 37232, USA.; ^13^ USAMRIID, Fort Detrick, MD 21702-5011, USA.; ^14^ BioPharmaceuticals R&D, AstraZeneca, Cambridge, CB21 6GH, UK.; ^15^ Vaccines and Immune Therapies, BioPharmaceuticals R&D, AstraZeneca, Gaithersburg, MD 20878, USA.

## Abstract

Despite the success of severe acute respiratory syndrome coronavirus 2 (SARS-CoV-2) vaccines, there remains a need for more prevention and treatment options for individuals remaining at risk of coronavirus disease 2019 (COVID-19). Monoclonal antibodies (mAbs) against the viral spike protein have potential to both prevent and treat COVID-19, and reduce the risk of severe disease and death. Here, we describe AZD7442, a combination of two mAbs, AZD8895 (tixagevimab) and AZD1061 (cilgavimab), that simultaneously bind to distinct, nonoverlapping epitopes on the spike protein receptor binding domain to neutralize SARS-CoV-2. Initially isolated from individuals with prior SARS-CoV-2 infection, the two mAbs were designed to extend their half-lives and reduce effector functions. The AZD7442 mAbs individually prevent the spike protein from binding to angiotensin-converting enzyme 2 receptor, blocking virus cell entry, and neutralize all tested SARS-CoV-2 variants of concern. In a nonhuman primate model of SARS-CoV-2 infection, prophylactic AZD7442 administration prevented infection, whereas therapeutic administration accelerated virus clearance from lung. In an ongoing phase 1 study in healthy participants (NCT04507256), a 300 mg intramuscular injection of AZD7442 provided SARS-CoV-2 serum geometric mean neutralizing titers greater than 10-fold above those of convalescent serum for at least 3 months, which remained 3-fold above those of convalescent serum at 9 months post-AZD7442 administration. Approximately 1 to 2% of serum AZD7442 was detected in nasal mucosa, a site of SARS-CoV-2 infection. Extrapolation of the time course of serum AZD7442 concentration suggests AZD7442 may provide up to 12 months of protection and benefit individuals at high-risk of COVID-19.

## Teaser

AZD7442 neutralizes SARS-CoV-2, has prophylactic and therapeutic efficacy in NHPs, and is projected to contribute to long-term human protection.

## INTRODUCTION

The coronavirus disease 2019 (COVID-19) pandemic caused by severe acute respiratory syndrome coronavirus 2 (SARS-CoV-2) continues to cause substantial morbidity and mortality worldwide. Although rollout of effective COVID-19 vaccines has reduced hospitalizations and death in several countries (*1*, *2*), SARS-CoV-2 infection continues to spread globally, as variants with increased transmissability and immune evasion continue to emerge. The need for new therapies to protect individuals who remain at risk of COVID-19 persists, which includes unvaccinated individuals, individuals who are unable to mount an adequate immune response following vaccination (*3*–*7*), and individuals with breakthrough infections despite full vaccination (*8*).

SARS-CoV-2-neutralizing monoclonal antibodies (mAbs) represent an approach for both the prevention and treatment of COVID-19 (*9*, *10*). The receptor binding domain (RBD) of the spike protein of SARS-CoV-2 mediates attachment to human angiotensin-converting enzyme 2 (ACE2) receptor, resulting in viral entry into host cells (*10*). Many individuals with SARS-CoV-2 infection develop neutralizing antibodies to the spike protein (*11*, *12*), which correlate with protection against symptomatic infection (*13*). Antibodies targeting the spike protein have also been shown to limit the progression of SARS-CoV-2 infection and the development of COVID-19 (*14*–*16*).

AZD7442 is a combination of two fully human, long-acting SARS-CoV-2-neutralizing antibodies, AZD8895 (tixagevimab) and AZD1061 (cilgavimab), in clinical development for the prevention of symptomatic COVID-19 and the treatment of mild-to-moderate and severe COVID-19 (*17*, *18*). AZD8895 and AZD1061 were derived from B cells isolated from individuals with prior SARS-CoV-2 infection (*19*). Their progenitor mAbs, COV2-2196 and COV2-2130, respectively, were shown to potently and synergistically neutralize SARS-CoV-2 in vitro and confer protection in animal models of SARS-CoV-2 infection when co-administered (*19*), supporting further development of this combination. The variable regions of the progenitor mAbs were reformatted as immunoglobulin 1 kappa (IgG1κ), with additional amino acid substitutions in the fragment crystallizable (Fc) regions to extend their serum half-lives (*20*, *21*) and reduce Fc gamma receptor (FcγR) and complement binding (*22*) to create AZD8895 and AZD0161. This study describes the preclinical and translational characteristics of AZD8895, AZD1061, and AZD7442, and evaluates the potential of AZD7442 to both prevent and treat SARS-CoV-2 infection in nonhuman primate (NHP) models. Additionally, we characterize AZD7442 pharmacokinetics and transudation to the nasal mucosa in healthy adult participants enrolled in a phase 1 clinical study.

## RESULTS

### AZD7442 antibodies, AZD8895 and AZD1061, simultaneously bind the SARS-CoV-2 spike protein with high affinity.

**Fig. 1.**
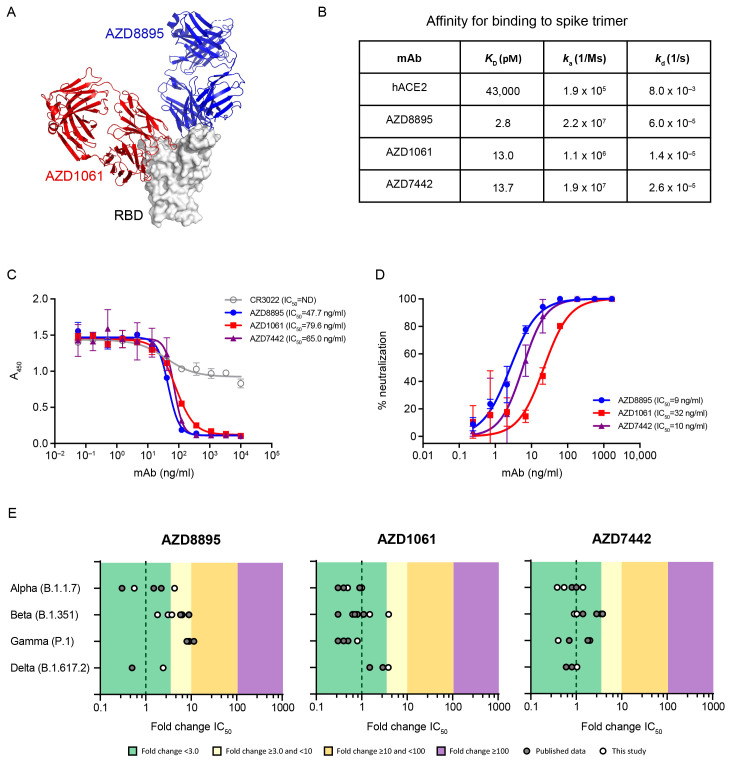
Simultaneous binding of AZD1061 and AZD8895 (AD7442) to the SARS-CoV-2 RBD blocks binding to ACE2 and potently neutralizes SARS-CoV-2 variants of concern. (**A**) AZD8895 and AZD1061 simultaneously bind to distinct, nonoverlapping epitopes on the spike protein RBD and sterically block RBD binding to ACE2. A side-view depiction shows cartoon representations of AZD8895 (blue) and AZD1061 (red) on top of the RBD (white) in surface representation based on co-crystal structures of AZD8895 and AZD1061 with RBD (*24*). (**B**) AZD8895, AZD1061, and AZD7442 binding kinetics on the SARS-CoV-2 S trimer (S2P ectodomain protein) are shown. Data shown are from a representative experiment from at least two independent assays (*58*). (**C**) AZD8895, AZD1061, and AZD7442 block binding of the RBD to ACE2. Measurements were taken across a series of mAb concentrations and the resulting nonlinear regression curves were used to calculate IC_50_ values. Data shown were performed in duplicate and represent at least two independent assays. (**D**) AZD8895, AZD1061, and AZD7442 neutralize the USA-WA1/2020 strain of SARS-CoV-2 in vitro. Nonlinear regression dose-response curves from a representative experiment are shown, with mean and SD error from two or more technical replicates. Mean IC_50_ values were calculated from three independent experiments. (**E**) AZD8895, AZD1061, and AZD7442 neutralize SARS-CoV-2 VOC in vitro. Data shown represent fold-change in neutralization potencies (IC_50_) of AZD8895, AZD1061 and AZD7442 against the Alpha (B.1.1.7), Beta (B.1.351), Gamma (P.1), and Delta (B.1.617.2) VOC as compared with the USA-WA1/2020 or AUS/VIC01/2020 reference strains. Data shown in solid circles have been published previously (*24*, *28*, *29*, *31*, *33*–*35*). Data shown in open circles are from this study, performed at Public Health England (Alpha, Beta, Gamma, and Delta), United States Army Medical Research Institute of Infectious Diseases (Alpha and Beta), and Integrated Research Facility, National Institute of Allergy and Infectious Diseases (Alpha and Beta). hACE2, human angiotensin-converting enzyme 2; IC_50_, half maximal inhibitory concentration; *k*_a_, association rate constant; *k*_d_, dissociation rate constant; *K*_D_, equilibrium dissociation constant; mAb, monoclonal antibody; RBD, receptor binding domain; SARS-CoV-2, severe acute respiratory syndrome coronavirus 2; SD, standard deviation; VOC, variant of concern.

Co-crystal structures of AZD8895 and AZD1061 showed simultaneous binding to the RBD at distinct, noncontiguous, and nonoverlapping epitopes **(****Fig. 1A****)**. Hydrogen-deuterium exchange mass spectrometry confirmed that AZD8895 and AZD1061 associate with distinct peptide residues on opposing faces of the RBD **(fig. S1)**. AZD8895, AZD1061, and AZD7442 bound to the SARS-CoV-2 spike protein with high affinity (equilibrium dissociation constant [*K*_D_] of 2.8, 13.0 and 13.7 pM, respectively). AZD7442 had a greater than 3,000-fold higher binding affinity compared to the binding affinity of the human ACE2 receptor (*K*_D_ of 43,000 pM) for the spike protein **(****Fig. 1B****)**. AZD8895 and AZD1061 potently blocked RBD binding to ACE2 individually (half maximal inhibitory concentration [IC_50_] 47.7 and 79.6 ng/ml, respectively; **Fig. 1C****),** demonstrating that the two mAbs can independently block RBD binding to ACE2.

### AZD7442 potently neutralizes SARS-CoV-2 variants of concern (VOCs) in vitro.

Since the COV2-2196 and COV2-2130 progenitor mAbs were re-engineered as IgG1κ formatted antibodies with Fc variant amino acids designed to confer extended antibody half-life (YTE modifications) and reduction of Fc receptor (FcR) interactions (TM modifications) to create AZD8895 and AZD1061, these were tested in neutralization assays to ensure that they retained their potency against the USA-WA/1/2020 strain and to evaluate their potency against emergent VOCs. The high binding affinities of the AZD7442 antibodies for the viral spike protein translated into potent SARS-CoV-2 neutralization activity. AZD8895, AZD1061, and AZD7442 potently neutralized the USA-WA1/2020 strain of SARS-CoV-2 (IC_50_ 9, 32, and 10 ng/ml, respectively; **Fig. 1D****)**. Importantly, as a result of a combination of two mAbs with nonoverlapping epitopes, AZD7442 retained potent neutralizing activity (fold-change IC_50_ <3.0) against SARS-CoV-2 Alpha, Beta, Gamma, and Delta VOCs compared with the USA-WA/1/2020 or AUS/IC01/2020 reference strains in the SARS-CoV-2 neutralization assays **(****Fig. 1E****)**.

### AZD7442 exhibits extended half-life in vivo and reduced Fc effector functions in vitro

**Fig. 2.**
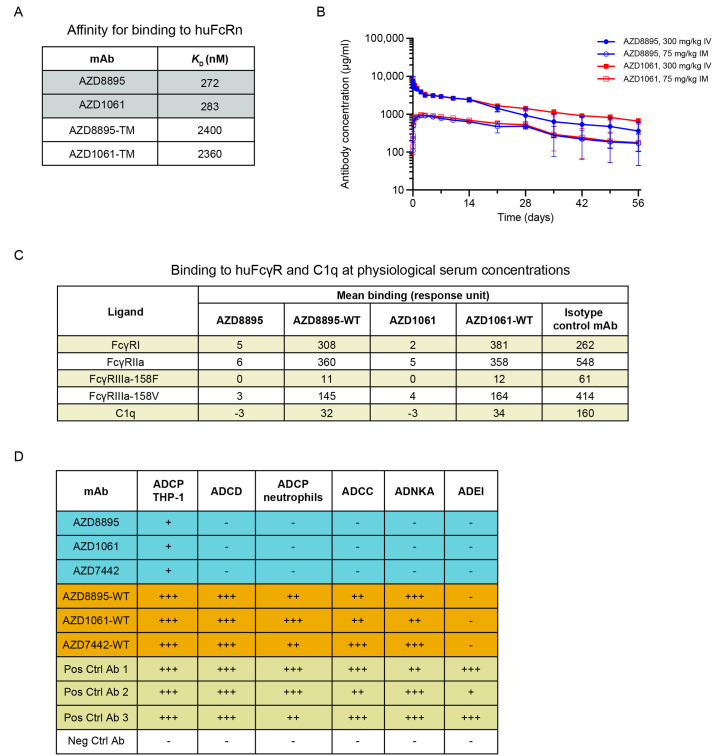
AZD7442 shows extended half-life and reduced Fc effector function in vivo. (**A**) AZD8895 and AZD1061 binding affinities for human FcRn are shown, measured by SPR with mAbs immobilized and titrated binding of huFcRn at pH 6.0. (**B**) AZD8895 and AZD1061 exhibited extended in vivo serum half-lives in NHPs. Data shown are mean ± SD. (**C**) AZD8895 and AZD1061 exhibited reduced binding to FcγR and C1q at physiological serum mAb concentrations. Isotype control = R347-WT, an antibody to HIV glycoprotein gp120 with no TM or YTE modifications in the Fc. Binding response = mAb binding response / ligand binding to probe (background subtraction). (**D**) AZD8895, AZD1061, and AZD7442 exhibited reduced Fc effector functions as compared with WT mAbs. Ratings were based on normalized AUC values with –, +, ++, and +++ assigned to values <25%, ≥25-50%, ≥50-75%, and ≥75% AUC, respectively. Negative control is an antibody to Ebola virus glycoprotein with no TM or YTE modifications in the Fc. Positive controls are two antibodies to SARS-CoV-2 spike protein RBD with no TM or YTE modifications in the Fc (Pos. Ctrl 1 and 2) and their combination (Pos. Ctrl 3). Ab, antibody; ADCC, antibody-dependent cellular cytotoxicity; ADCD, antibody-dependent complement deposition; ADCP, antibody-dependent cellular phagocytosis; ADEI, antibody-dependent enhancement of infection; ADNKA, antibody-dependent natural killer cell activation; AUC, area under the concentration curve; Fc, fragment crystallizable; FcγR, Fc gamma receptor; huFcRn, human neonatal Fc receptor; IM, intramuscular; IV, intravenous; *K*_D_, equilibrium dissociation constant; mAb, monoclonal antibody; Neg Ctrl, negative control; Pos Ctrl, positive control; SD, standard deviation; SPR, surface plasmon resonance; TM, substitution L234F/L235E/P331S in the antibody Fc region; WT, wild-type antibody (no substitution in Fc region).

AZD7442 includes YTE and TM modifications in the antibody Fc regions. In vitro, AZD8895 and AZD1061 exhibited approximately 9-fold greater affinities for the human neonatal Fc receptor (huFcRn) than AZD8895-TM and AZD1061-TM (mAbs with TM but not YTE substitutions; *K*_D_ 272, 283, 2,400 and 2,360 nM, respectively; **Fig. 2A****)**. This increased affinity for FcRn translated into extended half-lives (t_1/2_) for AZD8895 and AZD1061 in NHPs compared with mAbs without YTE modifications following intravenous (IV) administration of 600 mg/kg AZD7442. AZD1061 and AZD8895 had a median t_1/2_ of 19 days based on pharmacokinetic data collected over 8 weeks compared with t_1/2_ of 8 to 10 days for IgG in NHPs (*23*) **(****Fig. 2B****)**. AZD8895 and AZD1061 at physiological serum concentrations demonstrated little or no binding to tested FcγRs or C1q compared with AZD7442 antibodies with wild-type (WT) Fc (no YTE or TM substitutions; **Fig. 2C****)**. In vitro assays further confirmed that the AZD7442 antibodies displayed little or no Fc effector function, including antibody-dependent cellular phagocytosis, antibody-dependent cellular cytotoxicity, antibody-dependent complement deposition, or antibody-dependent natural killer cell activation. AZD7442, AZD8895, and AZD1061 also did not mediate antibody-dependent enhancement of infection **(****Fig. 2D** **and fig. S2)**.

### AZD7442 prevents and treats SARS-CoV-2 infections in NHPs.

AZD7442 was evaluated in NHP models of SARS-CoV-2 infection in prophylaxis or treatment settings in two separate studies, one in rhesus macaques (**fig. S3, A** and **B**) and one in cynomolgus macaques (**fig. S3, C** and **D**). For each study, three to four NHPs were administered either a 40 mg/kg dose of isotype control antibody or AZD7442 at a dose ranging from 0.04 to 40 mg/kg by IV infusion 3 days prior to viral challenge (prophylaxis) or a 40 mg/kg dose of AZD7442 24 hours after viral challenge (therapeutic analysis). To evaluate the contribution of Fc effector function to viral clearance, one group from each study received AZD7442-YTE (with YTE but not TM modification) as either prophylaxis (4 mg/kg, rhesus macaque study) or treatment (40 mg/kg, cynomolgus macaque study). In the prophylaxis studies, AZD7442 serum concentrations increased proportionally with dose across the 0.04 mg/kg to 40 mg/kg dose range. AZD7442 concentrations in the serum translated into high concentrations of serum neutralizing antibody titers in rhesus macaques and in cynomolgus macaques. Geometric mean pseudovirus neutralization (Neut_50_) titers peaked at 4.5 and 6.4 log_10_ for rhesus macaques administered 4 and 40 mg/kg AZD7442, respectively **(fig. S4, A** and **B)**; geometric mean SARS-CoV-2 plaque reduction neutralization test (PRNT_50_) titers of 3.4 and 4.6 log_10_ were measured for cynomolgus macaques administered 4 and 40 mg/kg AZD7442, respectively **(fig. S4, C** and **D)**. Low neutralizing titers were detected in the isotype control-treated rhesus macaques beginning day 10 post-infection consistent with the development of an adaptive humoral immune response (**fig. S4, A** and **B**).

**Fig. 3.**
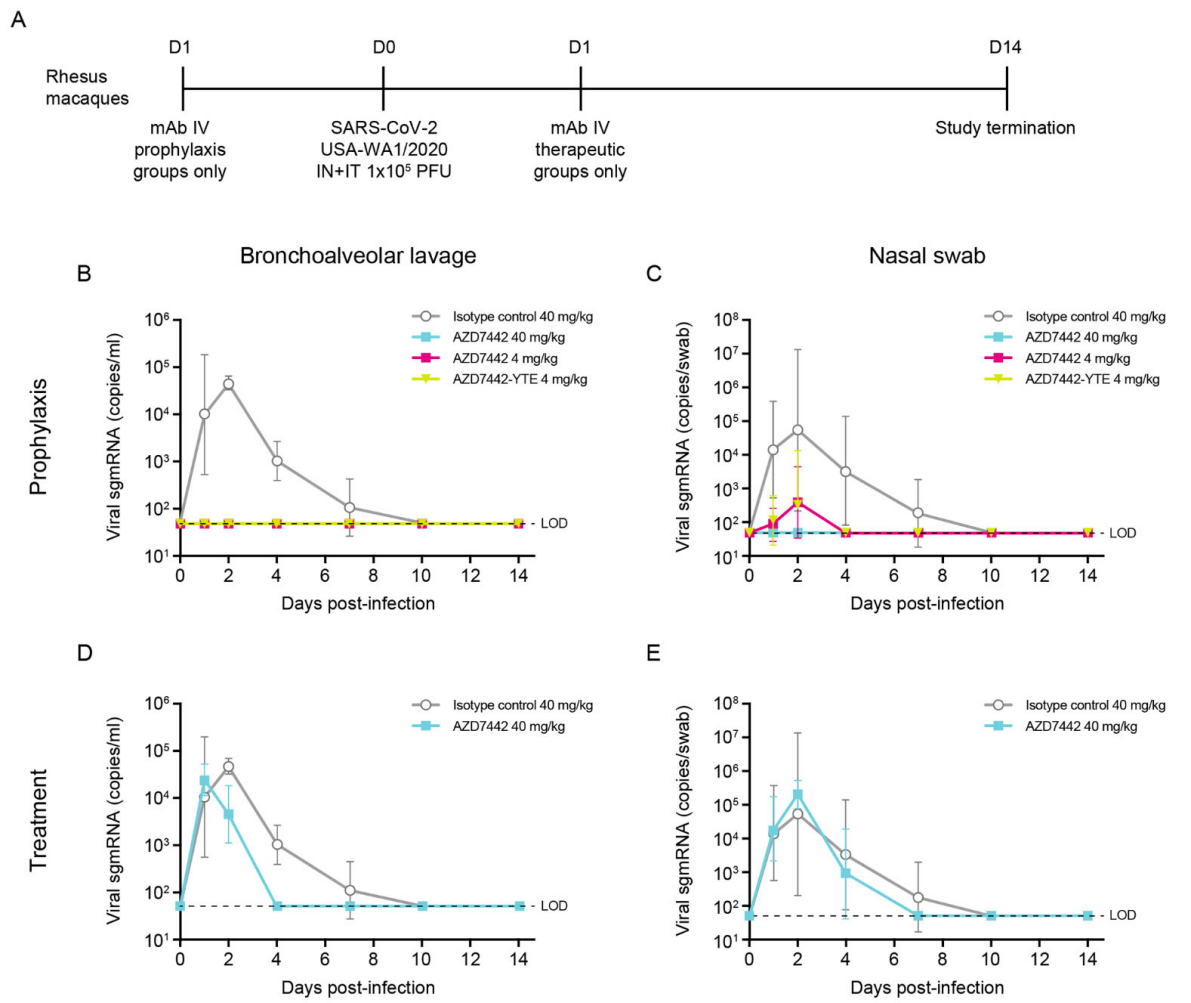
AZD7442 administration protects rhesus macaques against SARS-CoV-2 infection in prophylaxis or treatment settings. (**A**) A timeline of the in vivo SARS-CoV-2 challenge study is shown. Six-year-old rhesus macaques weighing 3.6 to 7.3 kg were used in this study. Rhesus macaques in prophylaxis groups received IV infusions of 40 mg/kg isotype control mAb R347-TM-YTE (n = 3), 40 mg/kg AZD7442 (n = 3), 4 mg/kg AZD7442 (n = 4), or 4 mg/kg AZD7442-YTE (n = 4), respectively, 3 days prior to challenge. Rhesus macaques in treatment group (n = 4) received an IV infusion of 40 mg/kg AZD7442 1 day after challenge. Rhesus macaques were challenged with 10^5^ PFU of SARS-CoV-2, split between IT and IN delivery on day 0. BAL and nasal swab samples were collected at days 0, 1, 2, 4, 7, 10, and 14. (**B**) Geometric mean ± SD viral burden are shown in BAL samples from rhesus macaques receiving isotype control mAb, AZD7442, or AZD7442-YTE as prophylaxis 3 days prior to SARS-CoV-2 challenge. (**C**) Geometric mean ± SD viral burden are shown in nasal swab samples from rhesus macaques receiving isotype control mAb, AZD7442 or AZD7442-YTE as prophylaxis 3 days prior to SARS-CoV-2 challenge. (**D**) Geometric mean ± SD viral burden are shown in BAL samples from rhesus macaques receiving isotype control mAb, AZD7442 or AZD7442-YTE as treatment 1 day after SARS-CoV-2 infection. (**E**) Geometric mean ± SD viral burden are shown in nasal swab samples from rhesus macaques receiving isotype control mAb, AZD7442 or AZD7442-YTE as treatment 1 day after SARS-CoV-2 infection. In (D) and (E), the arrow indicates the day of dosing relative to challenge on day 0. BAL, bronchoalveolar lavage; D, day; IN, intranasal; IT, intratracheal; IV, intravenous; LOD, limit of detection; mAb, monoclonal antibody; PFU, plaque forming unit; RNA, ribonucleic acid; sgmRNA, subgenomic messenger RNA; SARS-CoV-2, severe acute respiratory syndrome coronavirus 2; SD, standard deviation.

Rhesus macaques were challenged on day 0 with 10^5^ plaque forming units (PFUs) of SARS-CoV-2 strain USA-WA1/2020 **(****Fig. 3A****)**. SARS-CoV-2 viral subgenomic messenger RNA (sgmRNA) was measured by quantitative reverse-transcription polymerase chain reaction (qRT-PCR) in bronchoalveolar lavage (BAL) and nasal swab samples up to 14 days after virus challenge. In rhesus macaques treated prophylactically with isotype control, peak concentrations of viral sgmRNA were detected on day 2 post-infection, with geometric means of 4.66 log_10_ copies/ml (BAL) and 4.73 log_10_ copies/swab (nasal swab) of sgmRNA detected **(****Fig. 3, B** and **C****)**. In contrast, sgmRNA was undetectable in the BAL samples of rhesus macaques receiving AZD7442 or AZD7442-YTE **(****Fig. 3B****)**; low concentrations of sgmRNA were detected transiently (day 2 only) in nasal swab samples from two of four rhesus macaques receiving 4 mg/kg AZD7442 (3.32–3.60 log_10_ copies/swab) and from one of four animals that received 4 mg/kg AZD7442-YTE (4.94 log_10_ copies/swab) **(****Fig. 3C****)**. The undetectable concentrations of sgmRNA in the BAL samples from rhesus macaques that received as little as 4 mg/kg AZD7442 (comparable to the human 300 mg dose) indicates that prophylactic administration of AZD7442 can protect against SARS-CoV-2 lower respiratory tract infection in rhesus macaques. The lack of Fc effector function and complement binding did not impact efficacy in this model as the non-TM (AZ7442-YTE) and the TM-modified AZD7442 showed similar in vivo efficacies (**Fig. 3B** and **3C**). Rhesus macaques treated with AZD7442 24 hours after inoculation showed a reduction in viral sgmRNA 24 hours later (day 2 post-infection) in BAL (**Fig. 3D**) and in nasal swabs as compared to isotype control-treated NHPs (**Fig. 3E**). Importantly, AZD7442 administration resulted in accelerated viral clearance as detected in both BAL and nasal swabs within 4 and 7 days post-infection, respectively. In comparison, NHPs that received the isotype mAb did not fully clear the virus until day 10 post-infection **(****Fig. 3, D** and **E****)**.

**Fig. 4.**
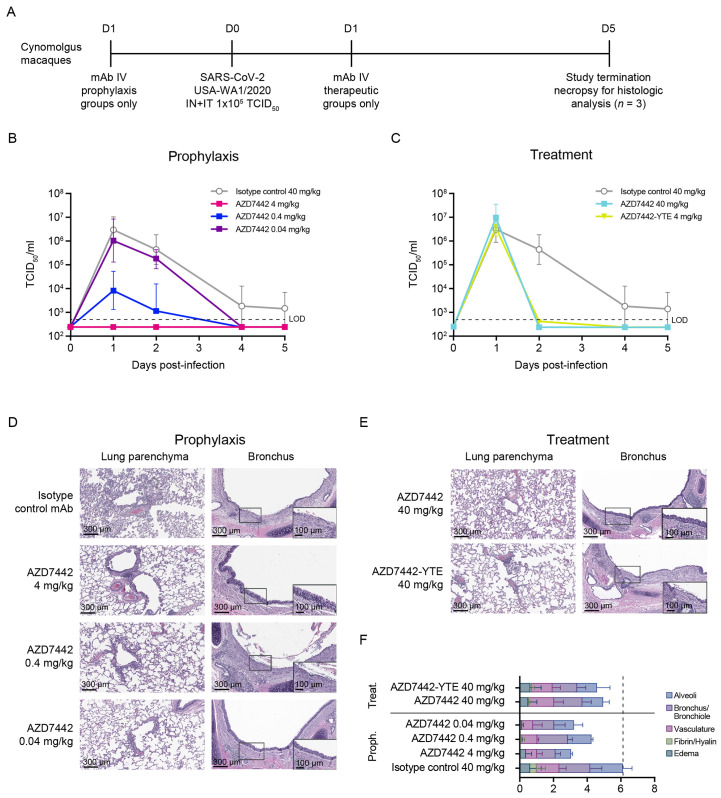
AZD7442 administration protects cynomolgus macaques against SARS-CoV-2 infection and associated lung immune pathology in prophylaxis and treatment. (**A**) A timeline of the in vivo SARS-CoV-2 challenge study is shown. 6-year-old cynomolgus macaques weighing 3.2 to 6.3 kg were used in this study. Cynomolgus macaques in prophylaxis groups (all groups n = 3), received IV infusions of 40 mg/kg isotope control mAb R347-TM-YTE, 40 mg/kg AZD7442, 4 mg/kg AZD7442, or 4 mg/kg AZD7442-YTE, respectively, 3 days prior to challenge. Cynomolgus macaques in treatment group (n = 3) received an IV infusion of 40 mg/kg AZD7442 1 day after challenge. Cynomolgus macaques were challenged with 10^5^ TCID_50_ of SARS-CoV-2, split between IT and IN delivery on day 0. BAL and nasal swab samples were collected at days 0, 1, 2, 4, and 5. Serum was collected on days -3, 0, 1, 2, 4, and 5. All animals were euthanized on day 5 post-infection for histopathology analyses. (**B**) Geometric mean ± SD viral burden are shown in BAL samples from cynomolgus macaques receiving isotype control mAb or AZD7442 as prophylaxis 3 days prior to SARS-CoV-2 challenge. (**C**) Geometric mean ± SD viral burden are shown in BAL samples from control cynomolgus macaques receiving isotype control mAb, or cynomolgus macaques receiving AZD7442 or AZD7442-YTE as treatment 1 day after SARS-CoV-2 infection. Arrow (↓) indicates day of dosing relative to challenge on day 0. (**D**) Lung histology following AZD7442 administration is shown for cynomolgus macaques receiving the indicated treatments. Hematoxylin/eosin-stained lung parenchyma and bronchus are shown from a representative animal (10x magnification). Inset shows 20x magnified image of bronchus. (**E**) A pathology score was assigned by board-certified veterinary pathologist based on histologic findings on eight lung sections per animal. Data are presented as mean + SD. The scores for one animal from the AZD7442 4 mg/kg prophylaxis dose group was excluded from analysis due to evidence of foreign material (plant matter) in multiple sections and observed inflammation inconsistent with SARS-CoV-2 infection. BAL, bronchoalveolar lavage; D, day; IN, intranasal; IT, intratracheal; IV, intravenous; LOD, limit of detection; mAb, monoclonal antibody; proph, prophylaxis; SARS-CoV-2, severe acute respiratory syndrome coronavirus 2; SD, standard deviation; TCID_50_, tissue culture infection dose; treat, treatment.

In the second study, cynomolgus macaques were challenged on day 0 with 10^5^ tissue culture infectious dose (TCID_50_) of SARS-CoV-2 strain USA-WA1/2020 **(****Fig. 4A****)**. SARS-CoV-2 burden was measured in BAL and nasal swab samples up to 5 days after virus challenge. Prophylactic AZD7442 administration demonstrated a dose-dependent reduction of infectious virus titers in BAL samples **(****Fig. 4B****)**, and a reduction in viral sgmRNA concentrations in BAL and nasal swab samples **(fig. S5, A** and **B)** compared with isotype control antibody. Importantly, the 4 mg/kg dose (comparable to the human 300 mg dose) was fully protective in both NHP studies. Consistent with observations from the rhesus macaque study, faster virus clearance was observed in cynomolgus macaques administered 40 mg/kg AZD7442 therapeutically compared with isotype control **(****Fig. 4C****)**. Of note, AZD7442 treatment reduced viral titers similarly to AZD7442-YTE (no TM modification) at an equivalent 40 mg/kg dose (**Fig. 4C**, and **fig. S5, C** and **D**), suggesting that Fc effector function and complement binding are not required for treating SARS-CoV-2 infection at this dose.

Lung sections from control cynomolgus macaques showed histological changes consistent with SARS-CoV-2 infection **(****Fig. 4D****)**. In sections of lung parenchyma, there was mild to marked perivascular cuffing of medium-caliber blood vessels by lymphocytes, alveolar wall thickening, and mixed inflammatory cell infiltrates comprising lymphocytes, neutrophils, and macrophages within alveolar spaces. There was mild to marked bronchial/bronchiolar inflammation with loss or blunting of bronchial/bronchiolar epithelium and infiltrates of lymphocytes and neutrophils within the lamina propria and submucosa. Although there was some variability between sections consistent with focal SARS-CoV-2 infection, lung sections from animals administered AZD7442 either prophylactically or therapeutically showed an overall reduction in inflammation compared with those treated with isotype control (**Fig. 4, D and E**). NHPs administered 4 mg/kg AZD7442 as prophylaxis had the fewest histological changes in the lung parenchyma and bronchial epithelium compared with lungs from NHPs administered the 0.4 or 0.04 mg/kg doses in prophylaxis, or the 40 mg/kg dose in treatment. Notably, lungs from NHPs treated with a 40 mg/kg dose of AZD7442-YTE (no TM) or AZD7442 post-infection had similar pathology scores **(****Fig. 4, E and F****)**, suggesting that AZD7442 with or without the TM modification at this dose provides equivalent protection against SARS-CoV-2-induced lung injury.

Taken together, these two NHP studies indicate that AZD7442 administration prophylactically protects NHPs against SARS-CoV-2 infection, and therapeutically accelerates viral clearance. Importantly, AZD7442 administration either prophylactically or therapeutically, reduced pulmonary inflammation and protected animals against alveolar damage associated with SARS-CoV-2 infection, suggesting AZD7442 can provide clinical benefit in both prevention and treatment settings.

### AZD7442 exhibits extended half-life and provides high anti*–*SARS-CoV-2 neutralizing antibody concentrations in healthy adult participants

The pharmacokinetic characteristics and transudation of AZD7442 to the nasal mucosa were evaluated in a randomized, double-blind, placebo-controlled, phase 1 study of 60 healthy adults (NCT04507256). This study is ongoing, and the data presented herein includes only the initial time points for which analyses have been completed. Participants in the active arm received an intramuscular (IM) dose of 300 mg AZD7442 (150 mg of each mAb administered sequentially), IV AZD7442 at doses of 300 mg, 1,000 mg, or 3,000 mg (150 mg, 500 mg, or 1,500 mg, respectively of each mAb administered sequentially), or 3,000 mg AZD7442 co-administered by IV (1,500 mg AZD8895 + 1,500 mg AZD1061); all groups included 10 healthy participants.

**Fig. 5.**
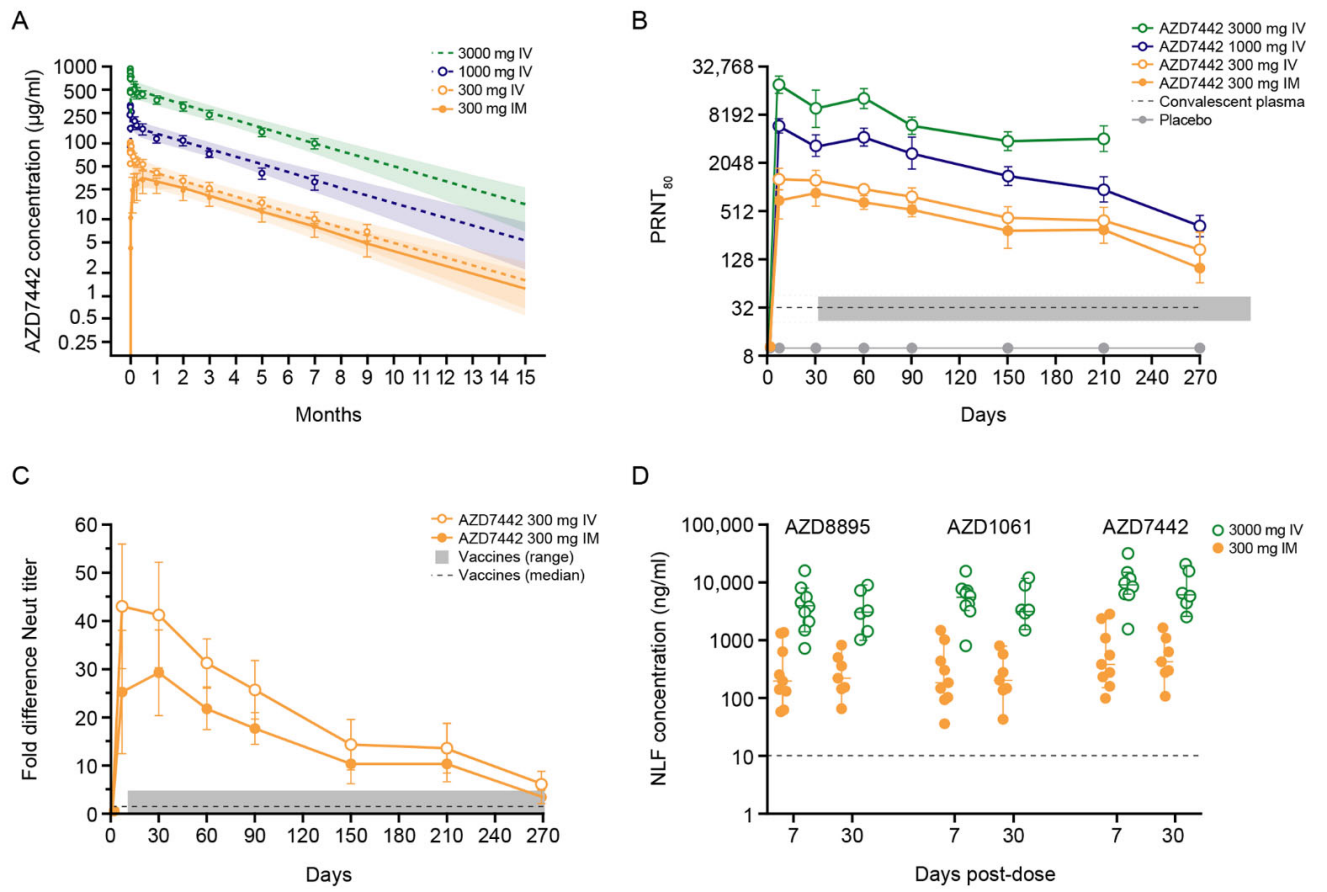
AZD7442 exhibits extended half-life, confers high anti-SARS-CoV-2 neutralizing antibody concentrations, and transudates to the mucosal epithelium in healthy adult participants. (**A**) Serum concentrations of AZD7442 were measured over 9 months following single IM or IV administration of AZD7442 in healthy participants. Symbols are observed mean ± SD and lines represent the predicted mean with shaded area representing the 90% prediction interval up to 15 months. (**B**) Geometric mean neutralizing antibody titers against SARS-CoV-2 are shown over 9 months following single IM or IV doses in healthy adult participants; data represent geometric mean PRNT_80_ titer ± SD for placebo or AZD7442 and GMT ± 95% CI for convalescent plasma samples (n = 28). (**C**) Fold-difference or ratio of SARS-CoV-2 neutralizing antibody titers to convalescent antibody titers for AZD7442 and vaccinated individuals is shown; vaccine range and median based on published data for seven different vaccines (*13*). (**D**) Concentrations of AZD8895, AZD1061 and AZD7442 (AZD8895 + AZD1061) were measured in the nasal lining fluid following 300 mg IM or 3000 mg IV doses of AZD7442. Graph shows individual and median concentration ± 95% CI. CI, confidence interval; IM, intramuscular; IV, intravenous; PRNT_50_, median plaque reduction neutralization titer; GMT, geometric mean neutralizing antibody titer; SARS-CoV-2, severe acute respiratory syndrome coronavirus 2; SD, standard deviation.

Serum concentrations of AZD8895 and AZD1061 were measured up to 9 months post dose. This confirmed the extended t_1/2_ of approximately 90 days for both antibodies in each dose cohort following either IV or IM administration **(****Fig. 5A****)**. After a single IM dose of 300 mg, the geometric mean C_max_ of AZD8895 (16.5 μg/ml) and AZD1061 (15.3 μg/ml) were similar and reached at a median T_max_ after 14 days. SARS-CoV-2 neutralizing antibody titers in serum conferred by AZD7442 were considerably higher than titers associated with convalescent plasma **(****Fig. 5B****)**. Geometric mean neutralizing titers afforded by AZD7442 300 mg IM and IV were approximately 22- and 41-fold higher, respectively, 7 days after dosing than those of convalescent serum samples. At day 270 post AZD7442 administration, the geometric mean neutralizing titers remained 3-fold higher than those of convalescent plasma samples (n = 28). To better understand the relative concentration of SARS-CoV-2 neutralizing antibodies conferred by AZD7442, we compared the ratio of neutralizing antibodies in serum following a single IM dose of 300 mg AZD7442 to neutralizing antibodies in convalescent serum samples. This analysis revealed that, within 1 week following administration, AZD7442 confers neutralizing antibody concentrations that were approximately 25-fold greater than those associated with convalescent serum (**Fig. 5C**). These concentrations remained 3-fold higher for 9 months (**Fig. 5C**). This ratio or fold-difference to convalescent serum was also compared to the ratios reported for seven COVID-19 vaccines (*13*). The concentration of neutralizing antibodies afforded by vaccines ranged from 0.5- to 4.0-fold, showing that neutralizing antibody concentrations afforded by AZD7442 remain at or above the range reported for vaccines up to 9 months (**Fig. 5C**).

To determine the proportion of AZD7442 that transudated to the mucosal surface, we measured the concentrations of AZD8895 and AZD1061 in nasal lining fluid (NLF). The median concentrations of AZD8895 and AZD1061 in NLF following an IM dose of 300 mg were 200 ng/ml through 30 days post-dose **(****Fig. 5D****)**. These concentrations correspond to 1 to 2% of those in serum, and suggest high abundance of neutralizing antibodies at the key site of infection.

## DISCUSSION

Antibody combinations comprising mAbs targeting distinct epitopes on the SARS-CoV-2 spike protein provide a higher threshold against virus escape from neutralization than single antibodies (*24*), as the virus would have to acquire mutations at both epitopes to escape neutralization. Structural analysis of the mAbs comprising AZD7442 (AZD8895 and AZD1061) showed that they simultaneously bind the RBD at distinct, nonoverlapping epitopes at the RBD-ACE2 interface (*24*). These data reinforce prior analyses of the AZD8895 and AZD1061 progenitor antibodies, COV-2196 and COV-2130, which demonstrated that they neutralize SARS-CoV-2 by blocking binding to ACE2 (*19*, *25*). Furthermore, we show that incorporation of the TM and YTE modifications in the Fc region did not affect binding to the RBD or potency of the antibodies to neutralize SARS-CoV-2 VOCs in vitro.

Mutations in the spike protein have been shown to increase viral infectivity and transmissibility (*26*, *27*), which threaten the effectiveness of vaccines and therapeutics by providing escape from neutralizing antibodies (*28*–*31*). mAb combinations that target distinct SARS-CoV-2 spike protein epitopes have demonstrated a higher threshold against virus escape than individual mAbs (*28*, *32*). AZD8895 and AZD1061 individually or in combination potently neutralized the USA-WA1/2020 strain of SARS-CoV-2. Importantly, we demonstrated in this study that AZD7442 retains potency to neutralize SARS-CoV-2 VOCs, including those with spike protein mutations that confer reduced susceptibility to AZD8895 or AZD1061 individually. Taken together, the data presented here and in other studies confirm that AZD7442 retains activity against all tested VOCs, including the Alpha (*24*, *28*–*30*, *33*), Beta (*24*, *29*–*31*, *33*), Gamma (*28*, *30*, *34*), and Delta (*24*, *35*) variants. While further testing is in progress for the Omicron VOC that emerged in November 2021, data published elsewhere shows that AZD7442 retains neutralizing activity against the Omicron variant, with reported IC_50_ values that range from 51 to 277 ng/mL (*36*–*39*).

In some viral infections, antibody-enhanced disease mediated by Fc engagement of immune cells and immune complex formation leading to enhanced immune activation has been proposed as a mechanism of exacerbation of immunopathology and inflammation (*40*). Although such a mechanism has not yet been established in SARS-CoV-2, the TM modification in the Fc region was introduced early in development of AZD7442 to reduce this potential risk by preventing FcγR and complement binding (*22*). This study confirmed that the introduction of the TM modification to AZD7442 abrogated Fc effector function without affecting neutralization activity. Clearance of SARS-CoV-2 was not affected by the TM modification and AZD7442 showed comparable protection to AZD7442-YTE (without TM modification) at 4 mg/kg in prophylaxis and 40 mg/kg in treatment in NHPs. Fc effector function has been shown to enhance viral clearance by some mAbs in mouse and hamster models of SARS-CoV-2 infection (*41*–*43*), but was not observed in NHPs in this study. This finding may be due to the differences between rodents and NHPs, rodent-adapted viral strains, doses of antibodies evaluated, or the targeting of different spike protein RBD epitopes of other mAbs. Although mAbs without TM modifications have demonstrated clinical benefit in the treatment of COVID-19 (*44*–*46*), the findings reported here demonstrated no substantial differences compared with AZD7442-YTE (without TM) and did not preclude ongoing clinical evaluation of AZD7442 with the TM modification. Indeed, recent results from a phase 3 clinical study of AZD7442 have demonstrated efficacy in the prevention of symptomatic COVID-19 (*47*), supporting the clinical potential of this extended half-life mAb combination with the TM modification. Ongoing studies of AZD7442 and other mAbs with or without Fc modifications may provide further insights into the clinical implications of the role of various Fc modifications, including TM, in the context of SARS-CoV-2 infection.

AZD7442 was also modified to include the half-life extending YTE substitutions (*20*, *21*). YTE-modified mAbs are under clinical evaluation in other infectious diseases, including for immunoprophylaxis against respiratory syncytial virus in infants (*48*). AZD8895 and AZD1061 have higher affinities for human FcRn at low endosomal pH than antibodies without the YTE modification, which translated into extended half-lives in NHPs and humans, consistent with other studies of YTE-enhanced mAbs (*20*, *49*, *50*).

At the 4 mg/kg dose, prophylactic AZD7442 administration prevented lower respiratory tract infection in NHPs as measured by viral sgmRNA or infectious virus titer in BAL samples. This is comparable with the prophylactic effect observed with other mAbs evaluated in similar NHP models (*51*, *52*). Viral sgmRNA was also largely undetectable in nasal swabs at this dose, suggesting that AZD7442 administration can prevent SARS-CoV-2 infection or virus shedding from the upper respiratory tract. The therapeutic administration of 40 mg/kg AZD7442 accelerated viral clearance as measured by viral sgmRNA concentrations or infectious virus titers in BAL and nasal swab samples. This finding contrasts with studies of remdesivir in rhesus macaques, where remdesivir reduced viral titers in BAL samples but not in the upper respiratory tract (*53*). Histologically, alveolar and bronchial sections of lungs from NHPs administered AZD7442 prophylactically or therapeutically all displayed reduced inflammation and pulmonary tissue damage associated with SARS-CoV-2 infection. Importantly, NHPs treated with the 4 mg/kg dose of AZD7442 exhibited serum antibody concentrations similar to those observed in individuals in the phase 1 study after they received a 300 mg IM dose of AZD7442, demonstrating the clinical relevance and translatability of the doses evaluated in these NHP models of SARS-CoV-2 infection.

Although NHP models do not mimic severe COVID-19 pathology in humans, the findings reported here contributed to the further clinical evaluation of AZD7442 in COVID-19 prevention and treatment settings. The 4 mg/kg AZD7442 IV dose that demonstrated prevention of SARS-CoV-2 infection in NHPs resulted in serum concentrations comparable to those achieved with an IM dose of 300 mg AZD7442 delivered to healthy adult human participants while animals that received 40 mg/kg AZD7442 as treatment showed near maximal serum concentrations within 1 day of dosing. The IM 300 mg dose of AZD7442 conferred an anti*–*SARS-CoV-2 geometric mean neutralizing antibody titer (GMT) at day 270 post-dose that was approximately 3-fold greater than those detected in convalescent plasma and similar to titers following vaccination. Given the high efficacy of vaccines in preventing COVID-19, these data for AZD7442 at this dose are encouraging. In addition, pharmacologically active concentrations of AZD7442 in the nasal mucosa at 7 and 30 days post-dose were, on average, 40-fold above the IC_50_ of AZD7442. These data support the potential of an IM dose of 300 mg AZD7442 to provide protection against COVID-19 for up to 12 months, but this requires confirmation in clinical studies.

Our report has several limitations. Both the cynomolgus and rhesus macaque models do not adequately mimic SARS-CoV-2 infection and disease in humans. Notably, a high dose of 10^5^ PFU or 10^5^ TCID_50_ is required to infect the animals, and peak viral load occurs earlier than in humans (day 1 to 2 versus day 4 to 7) (*54*). NHPs also do not present with severe COVID-19 disease or lung pathology associated with COVID-19. The limitations of the preliminary phase 1 data presented include the small number of participants in this analysis and a relatively short 6- to 9-month duration of follow-up for a half-life extended antibody that may be present in serum for up to 15 months. The phase 1 study enrolled healthy adults aged 18 to 55 years; the pharmacokinetic data presented may therefore not reflect the pharmacokinetic profile of populations more likely to utilize AZD7442 in the clinic, such as individuals with increased risk of severe COVID-19, including older individuals, individuals with comorbidities, and immunocompromised individuals. Further clinical data in these populations are expected from large scale phase 3 trials. Lastly, since these studies were conducted, another VOC, Omicron, has emerged and further studies are underway to identify the susceptibility of this variant to AZD1061, AZD8895 and AZD7442 neutralization.

In conclusion, AZD7442 demonstrated potent in vitro neutralization against SARS-CoV-2 VOCs, in vivo efficacy in both prevention and treatment settings of SARS-CoV-2 infection in NHPs, and extended half-life in NHPs and healthy adult participants. Pharmacokinetic predictions suggest that AZD7442 could provide up to 12 months of protection from COVID-19. Together, these findings supported the clinical study of AZD7442 as passive immunoprophylaxis to provide rapid protection in unvaccinated individuals or bolster the immune response of individuals who respond sub-optimally to vaccination, such as those with hematologic malignancies (*7*) or solid organ transplant recipients (*6*). Accelerated viral clearance and reduced immunopathology in NHPs also supported the study of AZD7442 as treatment for COVID-19 to prevent disease progression. A comprehensive AZD7442 clinical trial program is underway to address these important clinical questions; a phase 3 study demonstrated efficacy in the prevention of symptomatic COVID-19 (NCT04625725) (*47*), and phase 3 treatment studies will assess the role of AZD7442 across the spectrum of COVID-19 severity, including outpatient treatment of mild COVID-19 to prevent progression to severe disease (NCT04723394) and inpatient treatment of severe COVID-19 (NCT04501978 and NCT04315948), the results of which are eagerly anticipated.

## MATERIALS AND METHODS

### Study Design

In vitro studies were conducted to characterize AZD8895 and AZD1061 individually and in combination as AZD7442 to determine their affinity for binding to spike protein, their mechanism of action (to block spike protein binding to the ACE2 receptor), and potency to neutralize SARS-CoV-2 and variants. The mAbs were also assessed for binding to Fc receptors and effector function in comparison with control mAbs that lack the TM or YTE modifications in the Fc region. Assay details are provided in the Supplementary materials.

Pharmacokinetics of AZD7442 were assessed in NHPs (cynomolgus macaques) as part of an 8-week good laboratory practice toxicology study conducted at Charles River Laboratories, under protocols approved by the Charles River – Nevada Institutional Animal Care and Use Committee (IACUC). Animal numbers were determined taking into account ethical considerations, and the ability to detect potential toxicities in the different groups given the timepoints for necropsy. Animals were randomized and assigned to groups using a computer-based procedure prior to transfer to study; males and females were randomized separately. Personnel who performed ex vivo analyses for quantitation of AZD7442 were blinded to treatment groups at time of assay.

Prophylactic and therapeutic efficacy of AZD7442 was assessed in SARS-CoV-2 challenge studies in NHPs (rhesus macaques and cynomolgus macaques). These studies were approved by the IACUC (protocol numbers: 20-035 and 21-018P) and conducted at BIOQUAL, Inc., in adherence of the following standards of the Association for Assessment and Accreditation of Laboratory Animal Care: the 8^th^ edition of the *Guide for the Care and Use of Laboratory Animals*; the *Animal Welfare Act*; and the *2015 reprint of the Public Health Service Policy on Human Care and Use of Laboratory Animals*. The total number of animals used, group sizes and number of groups were considered the minimum required to properly characterize the effects of AZD7442, AZD7442-YTE and the isotope control mAb R347-TM-YTE, and the study was designed so that it did not need an unnecessary number of animals to accomplish its objectives. Animals were randomly assigned to groups based on weight and gender. Personnel who performed ex vivo analyses were blinded to treatment groups at time of assay. The assigned veterinary pathologist was blinded to treatment groups at time of slide reading. Animal Research: Reporting of In Vivo Experiments (ARRIVE, 2.0) guidelines were also followed in regard to the performance of all animal studies. Interventions to reduce pain, suffering and distress were taken whenever possible in accordance with these protocols.

Clinical results are reported from an ongoing phase 1, randomized, double-blind placebo-controlled, dose-escalation study of the safety, tolerability, and pharmacokinetics of AZD7442 in healthy adult participants with no prior history of COVID-19 and no prior receipt of a COVID-19 vaccine (NCT04507256). Participants, investigators, clinical staff, and the study monitor were all blinded from the assigned intervention. The study was conducted in compliance with the ethical principles originating in or derived from the Declaration of Helsinki and in compliance with the International Confederation on Harmonization Good Clinical Practice Guidelines. All participants provided written informed consent before entering the study. The study protocol and informed consent documentation were reviewed and approved by London – Riverside Ethics Committee, UK (reference: 20/LO/1010). Sixty healthy participants aged 18 to 55 years at a single site in the United Kingdom were randomized 5:1 to one of five dose cohorts to receive one of four AZD7442 doses (300 mg IM, 300 mg IV, 1,000 mg IV or 3,000 mg IV, each mAb administered sequentially at 50% of the total AZD7442 dose; 3,000 mg IV co-administration of AZD8895 and AZD1061 as a single mixed infusion) or placebo.

### NHP AZD7442 pharmacokinetic studies

Cynomolgus macaques were received from Orient BioResource and were between 2.2 and 5.1 years old; males weighing between 1.9 and 2.6 kg and females weighing between 2.2 and 2.5 kg at the time of dosing. AZD7442 was administered to cynomolgus macaques by an IV infusion at 600 mg/kg (n = 10) or by an IM injection at 300 mg/kg (n = 4); control animals were administered vehicle alone at volumes equivalent to AZD7442 by a single IV infusion (n = 12) or a single IM injection (n = 4). For the IV administered animals, all toxicology and safety assessment endpoints were evaluated at day 15 (n = 9 control animals and n = 6 AZD7442 animals) and day 57 (n = 4 for control and AZD7442 animals) necropsy timepoints.

The IM animals had only general in-life procedures, observations, and measurements collected. IV infusion was by a suitable peripheral vein using an infusion pump connected to a temporary indwelling catheter once for 15 min. AZD8895 and AZD1061 were administered by separate 15-min infusions, for a total infusion time of 30 min. A 1 ml saline flush was administered following each 15 min infusion. IM injection to the anterior thigh was performed on temporarily restrained but not sedated animals. AZD8895 and AZD1061 were administered separately by two injections, one injection per thigh. In-life measurements included post dose and detailed clinical observations, injection site dermal scoring, body weights, food consumption, veterinary physical examinations, ophthalmic examinations, electrocardiography exams, neurologic examination, blood pressure and heart rate, respiratory rate and body temperature. Blood was collected by venipuncture at various times before AZD7442 administration and up to 8 weeks post dose to measure AZD8895 and AZD1061 serum concentrations and ex vivo pharmacodynamic evaluations. At study termination, animals were euthanized by IV injection of a commercially available veterinary euthanasia solution, followed by exsanguination.

### NHP SARS-CoV-2 challenge studies

NHPs were quarantined and allowed to acclimatize for at least 7 days at BIOQUAL, Inc. Upon initiation of SARS-CoV-2 challenge procedures, the NHPs were housed in HEPA-filtered microisolator caging units. Potential confounders were not controlled for. Cage-side observations were performed at least twice a day. Physical assessments of anesthetized NHPs included heart rate, body weight, and rectal temperature at the time of sedation. No NHPs were excluded a priori after none showed signs of illness or poor health. Veterinary care was provided by full-time veterinarians. NHPs received a primate diet (Purina, Monkey Diet Jumbo) ad libitum and were provided environmental enrichment by BIOQUAL, Inc, in accordance with the requirements of the Office of Laboratory Animal Welfare, the USA Animal Welfare Act, and the Guide for the Care and Use of Laboratory Animals.

For the first NHP SARS-CoV-2 challenge study, 14 rhesus macaques (*Macaca mulatta*) of Indian origin, aged 5 to 6 years and weighing between 3.6 and 7.3 kg, received prophylactic 10 ml IV infusions of the following mAbs 3 days prior to SARS-CoV-2 challenge: 40 mg/kg isotype control mAb R347-TM-YTE (two male, one female); 40 mg/kg AZD7442 (two males, one female); 4 mg/kg AZD7442 (two males, two females); or 4 mg/kg AZD7442-YTE (three males, one female). Four additional rhesus macaques weighing between 4.6 and 6.6 kg received a therapeutic 10 ml IV infusion of 40 mg/kg AZD7442 1 day after SARS-CoV-2 challenge (two males, two females).

In the second NHP SARS-CoV-2 challenge study, 18 cynomolgus macaques (*Macaca fascicularis*) of Cambodian origin, aged 4 to 5 years old and weighing between 3.2 and 5.6 kg, received prophylactic 10 ml IV infusions of the following mAbs 3 days prior to SARS-CoV-2 challenge: 40 mg/kg isotype control mAb R347-TM-YTE (one male, two females); 4 mg/kg AZD7442 (one male, two females); 0.4 mg/kg AZD7442 (one male, two females); or 0.04 mg/kg AZD7442 (two males, one female). Six additional cynomolgus macaques weighing between 3.4 and 6.3 kg received therapeutic 10 ml IV infusions of 40 mg/kg AZD7442 (two males, one female) or 40 mg/kg AZD7442-YTE (two males, one female) 1 day after SARS-CoV-2 challenge.

All NHPs were challenged with SARS-CoV-2 strain USA-WA1/2020 on day 0: 10^5^ PFU (rhesus macaques) or 10^5^ TCID_50_ (cynomolgus macaques). Rhesus macaques were challenged with an in-house generated stock, lot no. 022320-1100, expanded in Vero E6 cells from BEI Resources (cat. no. NR-52281, lot no. 70033175) (*55*). Cynomolgus macaques were challenged with a stock obtained from BEI Resources (cat. no. NR-53872, lot 70040665) at a concentration of 6.9 × 10^4^ TCID_50_ per ml in Vero E6 cells. Virus inoculum was prepared on the day of administration at BIOQUAL, Inc., by research associates. SARS-CoV-2 was administered to NHPs under ketamine sedation, with the virus inoculum split between intratracheal and intranasal routes.

Samples were collected under anesthesia. Blood was collected by femoral venipuncture into BD Vacutainer serum separator gel tubes for analysis of serum human IgG concentrations and neutralization titer measurements. BAL samples were collected using rubber feeding tubes inserted into the trachea for virologic analyses. Nasal swab samples were collected using flocked swabs (COPAN Diagnostics Inc) for virologic analyses. One rhesus macaque from each of the four groups (40 mg/kg isotype control, 4 mg/kg prophylactic AZD7442, 4 mg/kg prophylactic AZD7442-YTE, or 40 mg/kg therapeutic AZD7442) was euthanized on day 2 post-infection for histopathology analyses. Cynomolgus macaques from all groups were euthanized on day 5 post-infection for histopathology analyses. The design for each study is summarized in **Fig. 3A** and **Fig. 4A**.

### Quantitation of NHP SARS-CoV-2 genomic RNA and sub-genomic mRNA by qRT-PCR

RNA was extracted from BAL and nasal swab samples using a QIAcube HT (Qiagen) and the Cador pathogen HT kit per manufacturer’s instructions and eluted in nuclease-free water. RNA was reverse transcribed using superscript VILO (Thermo Fisher Scientific) per manufacturer’s instructions and tested in duplicate with QuantStudio 6 and 7 Flex RTPCR System (Thermo Fisher Scientific). Viral titers were calculated to give copies/ml using a viral RNA standard curve and primers and probes (Integrated DNA Technologies, Inc.) that target the SARS-CoV-2 E gene sub-genomic mRNA (sgmRNA): genomic RNA - 2019-nCoV_N1-F:5′-GAC CCC AAA ATC AGC GAA AT-3′ ; 2019-nCoV_N1-R: 5′-TCT GGT TAC TGC CAG TTG AAT CTG-3′; and 2019-nCoV_N1-P: 5′-FAM-ACC CCG CAT TAC GTT TGG ACC-BHQ1-3′: sgmRNA SG-F: CGATCTTGTAGATCTGTTCCTCAAACGAAC; SG-R: ATATTGCAGCAGTACGCACACACA; and FAM-ACACTAGCCATCCTTACTGCGCTTCG-BHQ. To generate a standard curve for the SARS-CoV-2 E gene sgmRNA assay, the SARS-CoV-2 E gene sgmRNA was cloned into a pcDNA3.1 expression plasmid. This insert was transcribed using the AmpliCap-Max-T7 High Yield Message Marker Kit (Cellscript) to obtain RNA. The limit of detection was 50 copies/ml.

### Quantitation of human IgG in NHP serum samples collected in the SARS-CoV-2 challenge studies

The concentrations of AZD7442, AZD7442-YTE, and control R347-TM-YTE in NHP serum samples were determined using an in-house enzyme-linked immunosorbent assay (ELISA) for human IgG against a known standard curve. Absorbance at 450 nm was recorded using a VersaMax or Omega microplate reader. Background-subtracted absorbance values for each standard was fit to a nonlinear, sigmoidal (4PL) curve using GraphPad Prism, and the ensuing curve was used to calculate concentrations of human IgG in each serum sample. Detailed ELISA methodology is provided in the Supplementary materials.

### NHP serum SARS-CoV-2 neutralization assay

A pseudovirus neutralization assay (*56*) was used as previously described to quantify neutralizing antibody titers in rhesus macaque serum samples. Pseudovirus was purified from HEK293T cells co-transfected with the psPAX2 packaging construct (AIDS Resource and Reagent Program), luciferase reporter plasmid pLenti-CMV PuroLuc (Addgene), and spike protein expressing plasmid pcDNA3.1-SARS-CoV-2 SΔCT. To determine serum neutralizing activity, heat-inactivated serum samples were incubated with pseudovirus before adding to HEK293T-human ACE2 cells for 48 hours, after which cells were lysed using the Steady-Glo Luciferase Assay (Promega). SARS-CoV-2 neutralization titers were defined as the sample dilution at which a 50% reduction in relative light units was observed relative to controls. All processes were carried out according to manufacturer’s instructions. Serum samples from cynomolgus macaques were evaluated using the SARS-CoV-2 plaque reduction neutralization test (PRNT) at Viroclinics Biosciences and BIOQUAL, Inc., respectively. A standard number of SARS-CoV-2 infectious units were incubated with serial dilutions of serum. After a 1 hour pre-incubation period of the serum mixtures, 100 μl of the mixture was added to the cells for 16 to 24 hours. After incubation, cells were formalin-fixed followed by incubation with a mAb targeting the viral nucleocapsid protein, followed by a secondary anti-human IgG HRP conjugate and KPL TrueBlue substrate. Images of all wells were acquired using an ImmunoSpot analyzer (Cellular Technology Limited), equipped with software to quantitate the nucleocapsid-positive cells (virus signal). The median neutralization titer (PRNT_50_) was calculated as described previously (*57*).

### NHP lung histology

Lungs were removed immediately after euthanasia. NHP lung tissues were fixed in 10% neutral buffered formalin and submitted in 70% ethanol to the histology laboratory at AstraZeneca. Paraffin-embedded lung samples were sectioned at 4 μM and stained with hematoxylin and eosin for evaluation by a board-certified veterinary pathologist.

### Phase 1 clinical study design

To determine the pharmacokinetics of AZD7442, serum samples were collected and analyzed pre-dose (all cohorts), during infusion and after infusion (IV cohorts only), at 8 hours post-dose and 1-, 3-, 5-, 7-, 14-, 30-, 60-, 90-, 150-, and 210 days post dose (all cohorts), and 270 days post dose (300 mg dosing cohorts only). To determine the SARS-CoV-2 neutralizing activity of AZD7442, serum samples were collected and tested pre-dose and 7-, 30-, 60-, 90-, 150-, and 210 days post dose (all cohorts) and 270 days post dose (300 mg dosing cohorts only). To determine AZD7442 concentrations in NLF, Nasosorption Fx·i (Mucosal Diagnostics) samples were collected 7 and 30 days post dose. Preliminary interim results of serum and NLF antibody concentrations, and neutralizing antibody titers are reported to confirm preclinical information for AZD7442. AZD7442 concentrations and neutralizing antibody titers in human serum were predicted beyond month 9 using a pharmacokinetic model. This consisted of a central blood and peripheral distribution compartment with first-order absorption from the injection site into the central compartment, and first-order elimination from the central compartment.

### Phase 1 clinical study serum SARS-CoV-2 neutralization assay

Phase 1 clinical trial samples were evaluated using the SARS-CoV-2 plaque reduction serum neutralization assay (PRNT) at Viroclinics Biosciences. A standard number of SARS-CoV-2 infectious units were incubated with serial dilutions of serum. After a 1 hour pre-incubation period of the virus, 100 μl of the mixture was added to Vero E6 cells for 16 to 24 hours. After incubation, cells were formalin-fixed followed by incubation with a mAb targeting the viral nucleocapsid protein, followed by a secondary anti-human IgG peroxidase conjugate (Thermo Fisher Scientific) and KPL TrueBlue substrate. Images of all wells were acquired by a CTL ImmunoSpot analyzer, equipped with software to quantitate the nucleocapsid-positive cells (virus signal). The 80% neutralization titer (PRNT_80_) was calculated as described previously (*57*). Convalescent plasma was obtained from 28 adults aged ≥18 years with a confirmed PCR-positive SARS-CoV-2 infection. At the time of collection, donors had been admitted to the hospital >20 days after symptom onset.

### Human nasal lining fluid pharmacokinetic assay

Qualified assays were used to measure the concentration of AZD7442 and urea in NLF eluent. NLF samples were collected by Nasosorption FX·i. To extract NLF, 300 μl of elution buffer (1.0 mg/ml bovine serum albumin, 1% NP-40 in 1x phosphate-buffered saline, pH = 7.4) was added to each synthetic absorptive matrix. Immediately upon elution, up to 60 μl of NLF eluant was used to measure the urea concentration with an enzymatic-colorimetric assay. Next, 180 μl of NLF eluant was subjected to immunocapture and enzymatic digestion treatments identical to the method described for serum sample analysis. The ratio of urea concentrations in NLF eluant and in serum were used for normalization of AZD7442 concentrations in the NLF to correct for differences in NLF sample volumes collected, and extraction efficiency.

### Bioanalytical assay to measure AZD7442 pharmacokinetics in NHP, human serum, or human NLF

Separate validated assays with similar methodologies were used to measure the concentrations of AZD7442 in cynomolgus macaque and human serum. A 20 μl sample was diluted and extracted with streptavidin magnetic beads coated with biotinylated RBD of SARS-CoV-2. The isolated analytes were subjected to denaturation, reduction, alkylation, and trypsin digestion. After digestion, the extract was fortified with stable isotope labeled peptide internal standard working solution. The final extract was analyzed by ultra-high performance liquid chromatography coupled with tandem mass spectrometry (UHPLC-MS/MS) with positive electrospray. A linear, 1/concentration^2^ weighted, least-squares regression algorithm was used to quantify unknown samples.

### Statistical methods

Descriptive statistics were used to present data. Peptide uptake during hydrogen-deuterium exchange is shown as mean (standard deviation [SD]) and measures of affinity for binding to spike trimer protein were based on at least two independent experiments. Nonlinear regression curves were used to calculate IC_50_ values for binding of RBD to human ACE2 (for ≥2 independent assays). Percent neutralization is shown as regression dose-response curves, with means (SD) from two technical replicates, and mean IC_50_ values calculated from three independent experiments. In vivo, half-life and effector function parameters for AZD8895, AZD1061, and AZD7442 were assigned based on normalized area under the concentration curve (AUC) values. In vitro, Fc effector function versus mAbs with TM substitutions are reported as mean response with background subtracted from 2 or more independent experiments, and as AUC. Human IgG serum concentrations and serum neutralizing antibody titers in NHPs are provided as geometric mean (SD) as are viral burden in bronchial lavage and nasal swab samples in NHPs. All histological evaluation was performed by a board-certified veterinary pathologist with individual pathology scores reported as mean (SD). In human serum, antibody concentrations following IV and IM administration are shown as mean (SD), predicted mean, and 90% prediction interval, with neutralizing antibody titers reported as geometric mean PRNT_80_ titer (SD; placebo) or GMT (95% confidence interval [CI]; convalescent plasma). Fold difference for AZD8895 and AZD1061 concentrations in NLF are shown as individual values and as mean (95% CI). GraphPad Prism software (versions 8.4.3 or higher) was used for data analysis and graph production.

## Supplementary Materials

### This PDF file includes:

Materials and Methods Figs. S1 to S5 References (*59*–*67*)

### Other Supplementary Material for this manuscript includes the following:

Data file S1

MDAR Reproducibility Checklist
