# Supplementary material for: The SARS-CoV-2 monoclonal antibody combination, AZD7442, is protective in non-human primates and has an extended half-life in humans
Source: Sci Transl Med. 2022 Jan 25:eabl8124. doi: 10.1126/scitranslmed.abl8124 (PMC8939769; doi:10.1126/scitranslmed.abl8124)
Supplement: Supplementary file 1 — Materials and Methods Figs. S1 to S5 References (59–67) [file scitranslmed.abl8124_sm.pdf]

Science Translational Medicine
AAAS

Supplementary Materials for

The SARS-CoV-2 monoclonal antibody combination, AZD7442, is protective in non-human primates and has an extended half-life in humans

Yueh-Ming Loo *et al.*

Corresponding author: Mark T. Esser, mark.esser@astrazeneca.com

DOI: 10.1126/scitranslmed.abl8124

The PDF file includes:

Materials and Methods
Fig. S1 to S5
References (*59–67*)

Other Supplementary Material for this manuscript includes the following:

Data file S1
MDAR Reproducibility Checklist

Supplementary Materials

Materials and Methods

Protein expression and purification

Codon-optimized genes were cloned into mammalian expression vectors. Monoclonal antibodies (mAbs) were IgG1 constructs with wild-type fragment crystallizable (Fc) or Fc variants containing TM and YTE substitutions. The YTE substitution (M252Y/S254T/T256E) in the Fc region of immunoglobulin G (IgG) has been shown to increase affinity for the human neonatal Fc receptor (huFcRn) at lower endosomal pH (*49*), promoting recirculation and thereby extending the elimination half-life ($t_{1/2}$) of mAbs in humans by 85 to 117 days (*20, 48, 59-61*). The TM substitution (L234F/L235E/P331S) in the Fc region of IgG has been shown to eliminate antibody-dependent cellular cytotoxicity (ADCC) and complement-dependent cytotoxicity in clinical settings (*22, 62-64*).

Severe acute respiratory syndrome coronavirus 2 (SARS-CoV-2) spike protein variant constructs were designed using the sequence of the Wuhan-Hu-1 (GenBank: MN908947.3) isolate as a reference. SARS-CoV-2 receptor binding domain (RBD) (residues 334 to 526) constructs were cloned with N-terminal CD33 leader sequences and C-terminal GSSG linkers, AviTags, GSSG linkers, and 8x HisTags. SARS-CoV-2 N-terminal domain (NTD, residues 16 to 305) was cloned with an N-terminal CD33 leader sequence and C-terminal GSSG linker and 8x HisTag. SARS-CoV-2 trimers were designed as previously described (*58, 65*). The human angiotensin converting enzyme 2 (*ACE2*) gene (residues 19 to 615) was cloned with an N-terminal CD33 leader

sequence and either a C-terminal GSSG linker, AviTag, GSSG linker and 8x HisTag, or a C-terminal GSSG linker and 8x HisTag. Proteins were expressed in FreeStyle 293X or 293F cells transfected with 293fectin (Thermo Fisher Scientific). mAbs were isolated from supernatants by affinity chromatography using MabSelect, or Protein A or Protein G columns (GE Healthcare), eluted with 0.1 M glycine at low pH, then dialyzed into phosphate-buffered saline (PBS). Other proteins were isolated by affinity chromatography using a HisTrap column followed by size exclusion column chromatography (GE Healthcare). Purified proteins were pooled and analyzed by gel electrophoresis, and further evaluated by size-exclusion chromatography coupled with multi-angle light scattering for some proteins. Protein biotinylation was performed enzymatically with BirA biotin protein ligase (Avidity) and confirmed by Western blot or 4'-hydroxyazobenzene-2-carboxylic acid reagent (Thermo Fisher Scientific).

Hydrogen-deuterium exchange mass spectrometry

125 μl spike protein stock (4.3 μM trimer) was added to 10 μl immunoglobulin G (IgG) stock (10 mg/ml) to give a molar ratio of 1:1.2 (spike protein: IgG) of either AZD1061 or AZD8895 and equilibrated for 30 minutes at room temperature. For the LEAP technologies hydrogen-deuterium exchange (HDX) method, 7.6 μl of protein sample (either 4.3 μM spike protein stock [control], or 1:1.2 spike protein:IgG sample) was added to 52.4 μl label buffer (10 mM potassium phosphate, pH 6.2) and incubated for either 1, 10, or 100 minutes at 20°C. After incubation, 50 μl of this sample was added to 50 μl quench solution (100 mM potassium phosphate, 200 mM tris[2-carboxyethyl]phosphine, 2 M guanidine hydrochloride, pH 2.3). 95 μl of the quenched sample was injected onto the HDX manager (50 μl loop volume). For the liquid

chromatography–mass spectrometry (LC-MS) method, online digestion was performed at 20°C using an online pepsin column (Waters, Enzymate) at 100 µl/minute for 4 minutes at about 4800 psi. After trapping, peptides were separated by a C18 analytical column at 40 µl/minute using a 5 to 35% linear methyl cyanide gradient over 6 minutes. The MS analyzer was a Thermo orbitrap fusion instrument operating in orbitrap detection mode at a resolution of 120 k. Data were processed using biopharma finder v3.0.62.11, with a signal to noise threshold of 256 and a mass error threshold of 15 ppm. HDX data were processed using HDExaminer version 2, excluding the N-terminal residue of each peptide due to back exchange. Structural heat maps were generated using positional averaging for visualising exchange data (PAVED) (*66*).

AZD7442 binding affinity assay

Kinetic rates of AZD1061 and AZD8895 binding to SARS-CoV-2 spike protein trimer and spike protein RBD were evaluated by surface plasmon resonance technology (Biacore T200). Affinity to SARS-Cov-2 spike protein trimer was determined by first immobilizing murine anti-his antibody to a net response of about 5,000 resonance units (RUs) onto a CM5 chip, then capturing SARS-CoV-2 trimer and measuring binding of titrated IgG and antibody fragment (Fab) of AZD8895, AZD1061, or AZD7442 (on for 100 seconds and off for 500 seconds at 50 µl/minute). ACE2 affinity to SARS-Cov-2 spike protein trimer was determined by immobilizing Strep-Tactin XT to a net response of about 2400 RUs onto a CM5 chip, then capturing about 250 RUs SARS-CoV-2 S protein trimer and measuring binding to titrated ACE2-Avi-His (on for 100 seconds and off for 200 seconds at 50 µl/minute). Collected data were fit to a one-site binding equation using Biacore software to obtain binding kinetics measurements.

SARS-CoV-2 receptor binding inhibition assay

A modified ELISA was conducted to measure mAb inhibition potencies for blocking RBD binding to ACE2. 96-well plates were coated overnight at 4°C with 100 µl/well of Penta-His antibody (Thermo Fisher Scientific) diluted in PBS (pH 7.0) to 1 µg/ml final concentration. Plates were washed with PBST, and wells then blocked with 250 µl casein for 1 hour at room temperature. Blocking buffer was removed from plates and ACE2-His was captured by addition of 100 µl/well ACE2-His diluted in casein to 1 µg/ml. Plates were incubated for 2 hours at room temperature. During this time, mAbs were serially diluted in casein, starting at a concentration of 20 µg/ml. The mAb dilutions were then mixed with an equal volume of RBD-Fc (diluted to 0.1 µg/ml) and the mAb-RBD mixtures incubated for 1 hour at room temperature. Plates were washed with PBST to remove unbound ACE2-His before addition of 100 µL/well of the mAb-RBD mixtures. After incubation for 1.5 hours at room temperature, unbound protein was removed by washing with PBST. Bound RBD-Fc was detected by the addition of 100 µl/well horseradish peroxidase (HRP)-conjugated goat anti-human IgG Fc antibody; diluted 1:55,000 in PBST. Plates were incubated for 1 hour at room temperature, washed again with PBST and then developed with the addition of 100 µl/well tetramethylbenzidine (TMB; Thermo Fisher Scientific). Plates developed for 5 minutes at room temperature before acid quenching with 50 µl/well 2 N H_2SO_4. Wells were measured for absorbance at 450 nm using an EnVision 2105 Multimode Plate Reader (Perkin Elmer). Absorbance values were fit to nonlinear regression curve fit (four parameters) using GraphPad Prism software (version 8.4.3). Sample additions for blocking, secondary, and development

steps were all performed by Multidrop (Thermo Fisher Scientific). The assay was performed twice in duplicate.

SARS-CoV-2 neutralization assays

SARS-CoV-2 reference strains (USA-WA1/2020 or AUS/VIC01/2020) and variants of concern (VOCs) (B.1.1.7, B.1.351, P.1, and B.1.617.2) were obtained from BEI Resources or isolated from nasopharyngeal swabs obtained and expanded in Vero E6 cells. All viruses were shown to be identical to the described genotype of circulating viruses by whole-genome sequencing. Microneutralization assays at Public Health England (PHE), United States Army Medical Research Institute of Infectious Diseases (USAMRIID), and Integrated Research Facility, National Institute of Allergy and Infectious Diseases (IRF/NIAID) were used to measure mAb potency by incubating infectious virus with serially diluted mAbs (AZD8895, AZD1061, and AZD7742). Virus-susceptible monolayers (Vero E6 cells) were exposed to the mAb-virus mixture. Following fixation, cells were immunostained with antibodies specific for SARS-CoV-2 nucleocapsid or spike protein RBD (Sino Biological). Infected cells were detected using either a HRP-labelled secondary antibody with KPL TrueBlue peroxidase substrate for visualization, or an Alexa Fluor 488-labeled secondary antibody with high content fluorescence imaging for visualization. Half maximal inhibitory (IC_{50}) concentrations were determined from non-linear regression analysis. Relative reduction in neutralization potencies (fold-change IC_{50}) of mAbs against VOCs was determined relative to reference strains tested in parallel.

Monoclonal antibody binding to human neonatal Fc receptor (FcRn), Fc-gamma receptor (FcγR) and complement C1q

Binding kinetics of mAbs to huFcRn, FcγRs, and complement C1q were assessed by surface plasmon resonance using a Biacore T200 (Cytiva). FcRn binding to the mAbs was measured at pH 6.0 and 7.4 and at 750 nM (2000 seconds at 5 µl/minute). The assay was performed in duplicate. Equilibrium dissociation constants (K_D) for mAb binding were determined at pH 6.0 by fitting data to a 1:1 binding isotherm equation using Biacore evaluation software (Cytiva). pH dependency was calculated as (1 – (RU pH 7.4 / RU pH 6.0)) × 100.

Human FcγRs was immobilized to a net response of 3000 to 8000 RU onto a CM5 sensor chip; C1q protein was immobilized to a net response of 5000 RU onto a CM4 chip. AZD8895, AZD8895-TM, AZD1061, and AZD1061-TM binding was measured at physiological mAb concentrations observed in human serum at about 50, 150, or 450 µg/ml (333, 1000, or 3000 nM; 2000 seconds at 10 µl/minute) with final response measurements at each sample dilution used to determine binding as a function of concentrations. Assays were performed in, and data were fit to a one site binding equation in GraphPad Prism.

Chemical conjugation of SARS-CoV-2 spike protein trimer to fluorescent polystyrene beads

Fluorescent polystyrene beads (Thermo Fisher Scientific) were washed in sterile cell culture-grade water and centrifuged at 15,000 × *g*. The beads were resuspended in 100 mM sodium phosphate buffer, pH 6.2. Sulfo-N-hydroxysulfosuccinimide (sulfo-NHS)

(Thermo Fisher Scientific) reconstituted at 50 mg/ml in sterile cell culture-grade water and 1-ethyl-3-[3-dimethylaminopropyl] carbodiimide reconstituted at 50 mg/ml in 100 mM sodium phosphate buffer (pH 6.2, activation buffer) were added to beads and vortexed thoroughly to mix. The beads were incubated at room temperature on a rotator. After incubation, the beads were centrifuged at 15,000 × *g* and the supernatant was discarded. The bead pellet was resuspended in 50 mM 2-(N-morpholino) ethanesulfonic acid (MES) coupling buffer pH 5.0, and vortexed thoroughly to mix. The beads were centrifuged at 15,000 × *g*, supernatant discarded, and washed in coupling buffer for a total of three washes. Beads were pelleted after final wash and resuspended in 50 mM MES buffer pH 5.0. Approximately 25 µg of SARS-CoV-2 spike protein trimer was added to the beads and incubated for 2 hours at room temperature on a rotator. Beads were centrifuged at 15,000 × *g* and resuspended in PBST (blocking/storage buffer) and incubated for 30 minutes at room temperature on a rotator. Beads were centrifuged at 15,000 × *g* and washed three times in PBST (wash buffer). After the final wash, beads were centrifuged at 15,000 × *g* and resuspended in PBS. The coupled fluorescent beads were stored at 4°C in the dark until use.

Antibody-dependent cellular phagocytosis with THP-1 cells

Antibody-dependent cellular phagocytosis (ADCP) assesses the ability of antibodies to induce phagocytosis of antigen-functionalized fluorescent beads by monocytes using Fc receptors. Fluorescent, streptavidin-conjugated polystyrene beads were coated with biotinylated SARS-CoV-2 spike protein trimer. Diluted antibody (in PBS) was added, and unbound antibodies were washed away. The antibody:bead complexes were added to undifferentiated THP-1 cells (American Type Culture Collection [ATCC]), a monocytic

cell line, and phagocytosis was allowed to proceed overnight. The cells were then washed and fixed, and the extent of phagocytosis was measured by flow cytometry to establish the number of fluorescent THP-1 cells and determine the degree of fluorescence. Data are reported as a phagocytic score, which considers the proportion of effector cells that phagocytosed (fluorescent cells) and the amount of phagocytosis (degree of fluorescence). The mAbs were tested for ADCP activity at a range of 5 μg/ml to 2.3 ng/ml.

Antibody-dependent cellular phagocytosis with neutrophils

ADCP with neutrophils (ADNP) assesses the ability of antibodies to induce the phagocytosis of antigen-coated targets by primary neutrophils. Fluorescent, streptavidin-conjugated polystyrene beads were coupled to biotinylated SARS-CoV-2 spike protein trimer. Diluted antibody (in PBS) was added, and unbound antibodies were washed away. The antibody:bead complexes were added to primary neutrophils isolated from healthy blood donors using negative selection (StemCell EasySep Direct Human Neutrophil Isolation Kit) per manufacturer's instructions, and phagocytosis was allowed to proceed for 1 hour. The cells were then washed and fixed, and the extent of phagocytosis was measured by flow cytometry as described above. Data are reported as a phagocytic score, which considers the proportion of effector cells that phagocytosed and the degree of phagocytosis. Each sample was run in biological duplicate using neutrophils isolated from two distinct donors. The mAbs were tested for ADNP activity at a range of 67 μg/ml to 30.6 ng/ml.

Antibody-dependent cellular cytotoxicity

Antibody-dependent cellular cytotoxicity (ADCC) tests the ability of antigen-specific antibodies to recruit natural killer (NK) cell lytic activity. Target cells were biotinylated (EZ-Link Sulfo-NHS-LC-Biotin) and stained with one of two dyes: half with CellTrace Violet and half with CellTrace Far Red (Thermo Fisher Scientific). The stained cells were then pulsed with either streptavidin-conjugated stabilized SARS-CoV-2 spike protein trimer or left unpulsed. Diluted antibody (in PBS) was added to a 1:1 mixture of the target cells stained with each dye. NK cells were purified from healthy blood donor leukopaks using commercially available negative selection kits (EasySep Human NK Cell Isolation Kit, StemCell) per manufacturer's instructions, and were added and incubated for 4 hours. Following this incubation, the cells were stained with a viability dye (Zombie Green Fixable Viability Kit, BioLegend). Lysis was measured by flow cytometry as described above and reported as the percentage of dead (viability dye positive) antigen-coated cells. The mAbs were tested for ADCC activity at a range of 25 μg/ml to 1.5 ng/ml.

Antibody-dependent complement deposition

Antibody-dependent complement deposition (ADCD) assesses the recruitment of complement component C3b on the surface of antigen-coupled beads. Neutravidin-conjugated polystyrene beads were coated with biotinylated SARS-CoV-2 spike protein trimer. Diluted antibody (in PBS) was added, and unbound antibodies were washed away. Commercially available guinea pig complement (Meridian Life Science; RRID:AB_151156) was then added as a source of complement. Following a brief incubation, the complement was washed away, and a fluorescein isothiocyanate (FITC)-

conjugated mAb specific for guinea pig C3 (Abcam) as added. Complement deposition was measured using flow cytometry as described above and was reported as the median fluorescent intensity of FITC. The mAbs were tested for ADCD activity at a range of 100 µg/ml to 4646 ng/ml.

Antibody-dependent enhancement of infection

The antibody-dependent enhancement of infection (ADEI) assay measures the capacity of antibodies to facilitate the infection of FCγR-expressing cells. Antibodies were diluted in cell culture medium, and SARS-CoV-2 spike protein-pseudotyped lentiviruses encoding firefly luciferase were added. The antibodies and viruses were incubated for 1 hour, after which Raji cells (a B cell line that naturally expresses FCγR2; ATCC) were added. The cultures were incubated for 24 hours, after which the culture medium was removed, and luciferase assay reagent was added. Following a 2-minute incubation to allow for cell lysis, the supernatant was transferred to a black 96-well plate, and luciferase activity was measured using a luminometer. Data are reported as the relative infection rate compared with infection in the absence of antibody. The mAbs were tested for ADEI activity at a range of 1 µg/ml to 0.125 µg/ml.

Antibody-dependent natural killer cell activation

Antibody-dependent natural killer cell activation (ADNKA) assesses antigen-specific antibody-mediated natural killer (NK) cell activation against protein-coated plates. Stabilized SARS-CoV-2 spike protein trimer was used to coat ELISA plates, which were then washed and blocked. Diluted antibody (in PBS) was added to the antigen-coated plates, and unbound antibodies were washed away. NK cells, purified from healthy

blood donor leukopaks using commercially available negative selection kits (StemCell EasySep Human NK Cell Isolation Kit) were added, and the abundance of the activation marker CD107a and intracellular cytokines (interferon-gamma [IFN-γ] and macrophage inflammatory protein-1β [MIP-1β]) were measured after 5 hours using flow cytometry as described above. Data are reported as the percentage of cells positive for each of the activation markers (CD107a, IFN-γ and MIP-1β). Each sample was tested with at least two different NK cell donors, with all samples tested with each donor. The mAbs were tested for ADNKA activity at a range of 20 µg/ml to 9.1 ng/ml.

Quantitation of human IgG in NHP serum by ELISA

ELISA plates were coated with anti-human IgG (Thermo Fisher Scientific) diluted in Dulbecco's phosphate-buffered saline (Thermo Fisher Scientific), incubated at 4°C overnight, washed in PBS and blocked in casein buffer. After incubation, casein buffer was removed and the plates blotted dry. Standard curves were prepared by creating two-fold dilutions of the respective mAbs in casein buffer. Serum samples were similarly diluted in casein buffer. Diluted standard or serum samples were applied to ELISA plates. One hour later, samples were removed and the plates washed in PBS before applying a 1:10,000 dilution of anti-human IgG HRP (Thermo Fisher Scientific). Following a 1 hour incubation in the dark at room temperature, plates were washed and developed with the addition of TMB SureBlue Start solution (SeraCare KPL). TMB Stop solution (SeraCare KPL) was added to each well to stop the reaction and absorbance at 450 nm recorded using a VersaMax or Omega microplate reader. Background-subtracted absorbance values for each standard was fit to a nonlinear, sigmoidal (4PL) curve using GraphPad Prism (version 8.4.3), and the ensuing curve was used to

calculate concentrations of human IgG in each serum sample.

Supplementary Figures

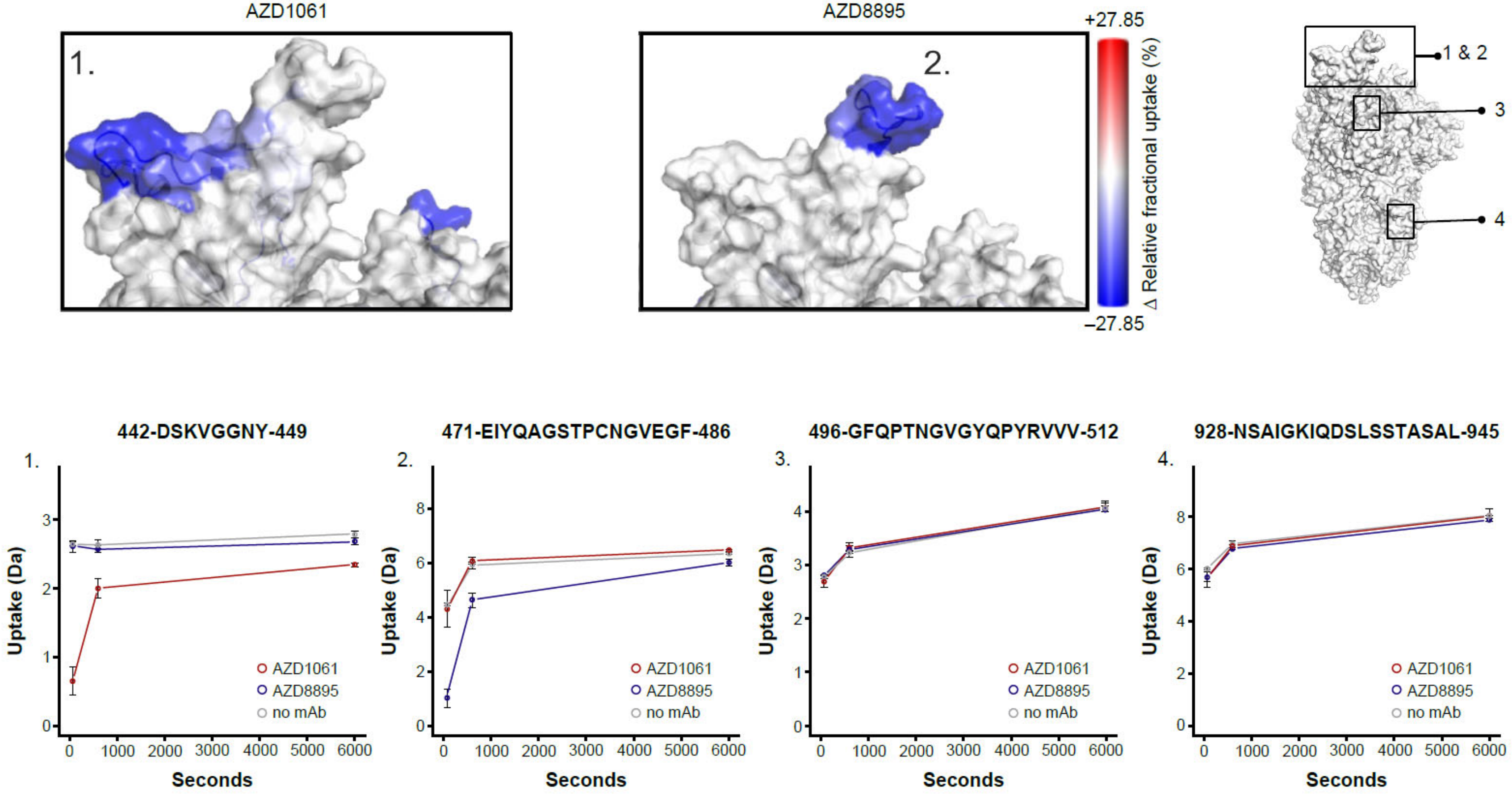

Figure S1. Hydrogen-deuterium exchange confirms that AZD8895 and AZD1061 bind distinct, non-overlapping sites on the SARS-CoV-2 spike protein RBD.

Structural heat map of HDX data reveals relative protection from HD exchange in distinct, complementary regions of the RBD following binding of AZD1061 (box 1, top left) or AZD8895 (box 2, top right). Below panels: example uptake plots of peptides covering these regions (numbered 1 and 2 for the sites of AZD1061 and AZD8895, respectively) as well as two other peptides distal to these sites showing similar deuterium uptake behavior (peptides 3 and 4, as highlighted on the spike protein structure in the top left) are shown for spike protein alone (cyan), spike protein + AZD1061 (black) and pike protein + AZD8895 (yellow). Error bars show standard deviation (SD), n=3. PDB 6Z97 (*67*). mAb, monoclonal antibody; RBD, receptor-binding domain; SARS-CoV-2, severe acute respiratory syndrome coronavirus 2; SD, standard deviation.

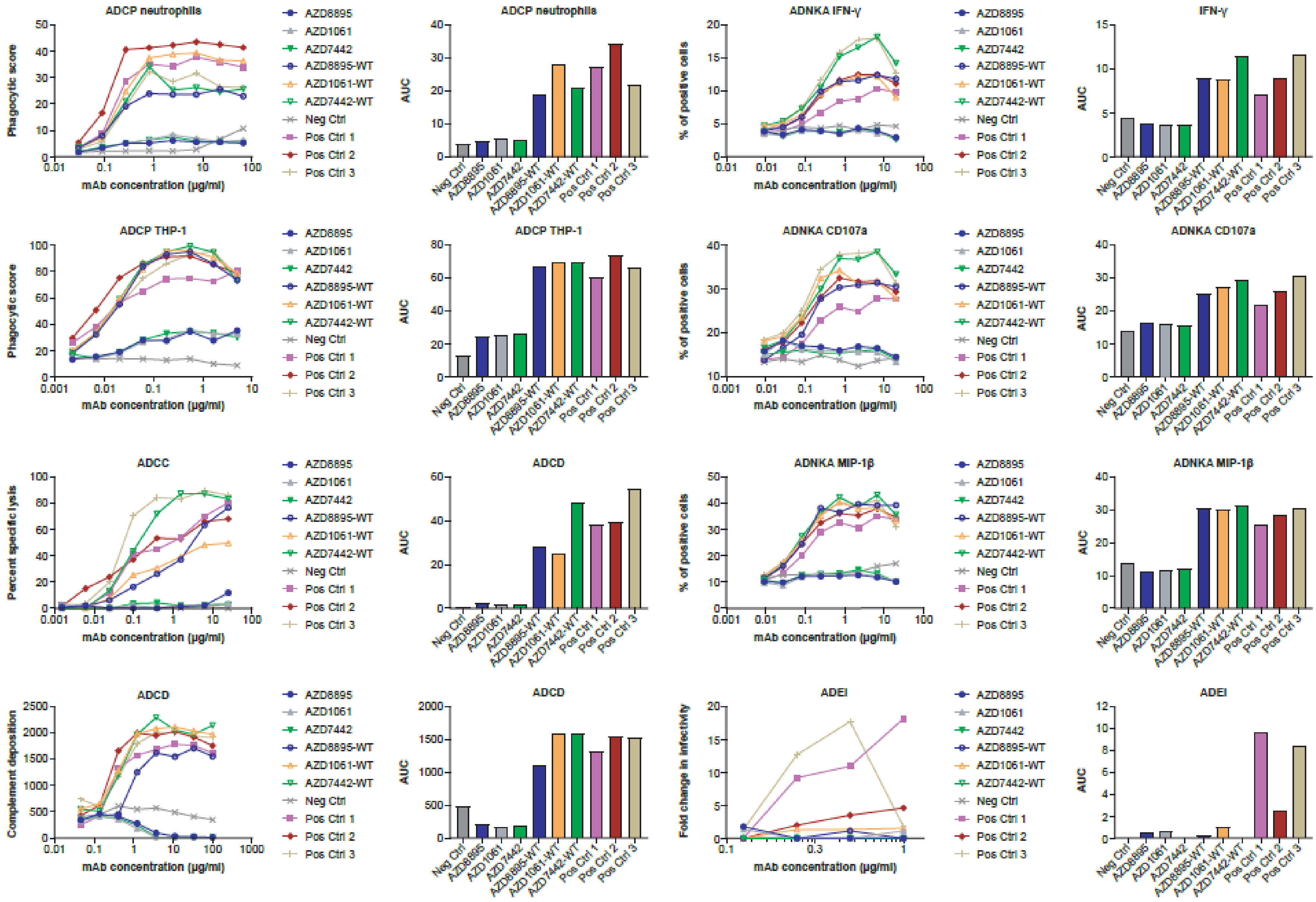
ADCP neutrophils
Phagocytic score
mAb concentration (µg/ml)
AZD8895
AZD1061
AZD7442
AZD8895-WT
AZD1061-WT
AZD7442-WT
Neg Ctrl
Pos Ctrl 1
Pos Ctrl 2
Pos Ctrl 3
AUC
ADNKA IFN-γ
% of positive cells
IFN-γ
ADCP THP-1
ADNKA CD107a
ADCC
Percent specific lysis
ADCD
ADNKA MIP-1β
Complement deposition
ADEI
Fold change in infectivity

Figure S2. In vitro assays confirm that AZD8895, AZD1061, and AZD7442 demonstrate reduced Fc effector function consistent with mAbs with TM substitutions.

In vitro ADCP, ADCC, ADNKA, ADCD, and ADEI assays were conducted with mAbs diluted across a range of concentrations. For ADCP assays, effector cells were measured for the uptake of spike protein-bearing fluorescent beads in the absence or presence of mAbs by flow cytometry. For ADCC assay, spike protein-expressing target cells were measured for neutrophil-mediated killing in the absence or presence of mAbs by flow cytometry. For ADNKA assay, primary NK cells were exposed to spike protein-coated plates in the absence or presence of mAbs and flow cytometry analyses conducted to measure expression of activation markers and cytokines. For ADCD assay, flow cytometry analyses were conducted to measure the deposition of complement proteins onto spike protein-coated beads in the absence or presence of mAbs. For ADEI assay, Raji cells were incubated with a spike protein-expressing pseudovirus that encoded a luciferase reporter. Incubations were performed in the absence or presence of mAbs with virus uptake measured by luciferase expression. Graphs show mean response subtracting background from 2 or more independent experiments. Bar charts show AUC analysis for each graph. ADCC, antibody-dependent cell cytotoxicity; ADCD, antibody-dependent complement deposition; ADCP, antibody-dependent cellular phagocytosis; ADEI, antibody-dependent enhancement of infection, ADNKA, antibody-dependent NK activation; AUC, area under the curve, CD, cluster of differentiation; Ctrl, control; Fc, fragment crystallizable; IFN-γ, interferon gamma; mAb, monoclonal antibody; MIP-1b, macrophage inflammatory protein also known as CCL4; Neg, negative; Pos, positive; THP-1, human monocytic cell line.

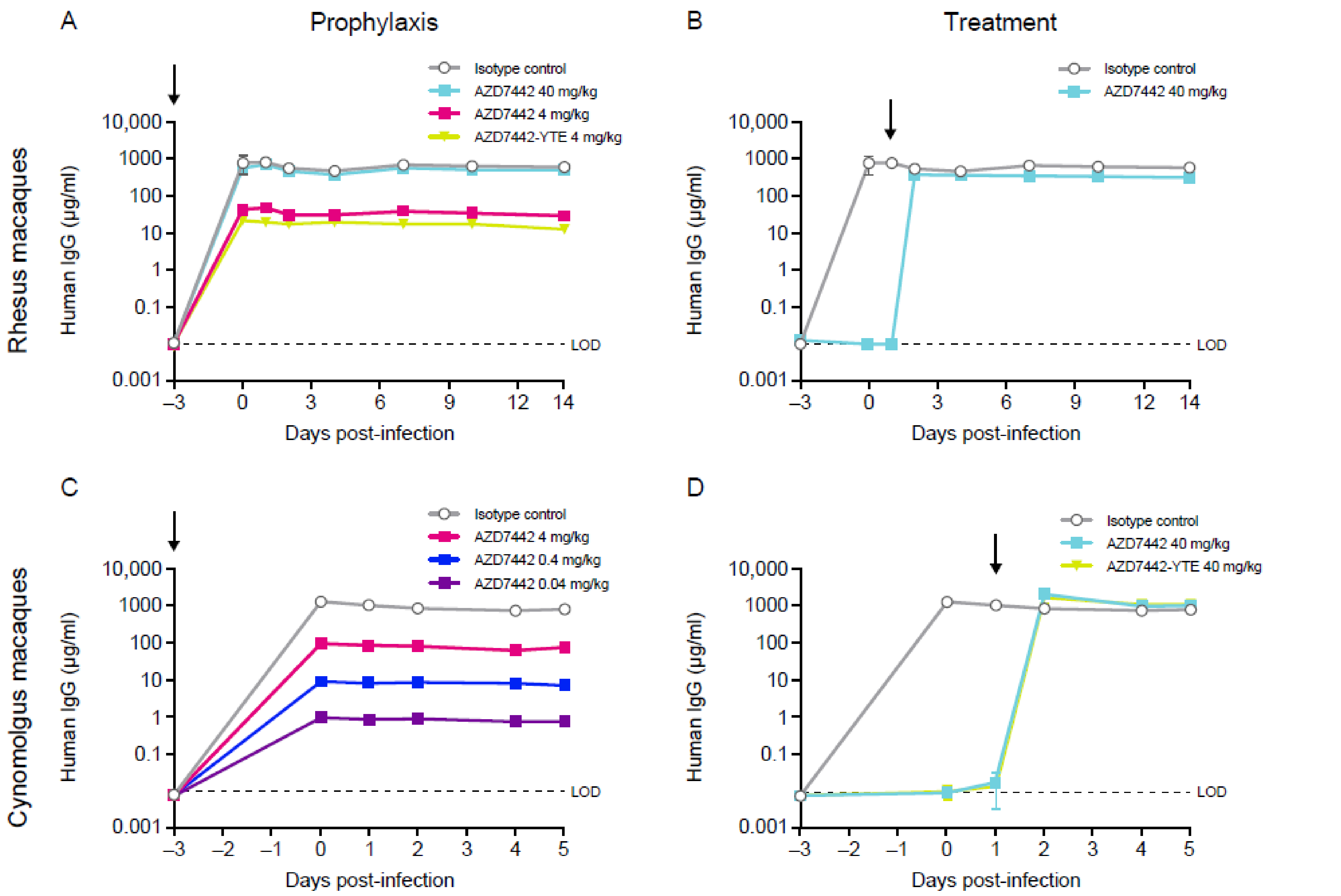
A
Prophylaxis
B
Treatment
Rhesus macaques
Human IgG (µg/ml)
Isotype control
AZD7442 40 mg/kg
AZD7442 4 mg/kg
AZD7442-YTE 4 mg/kg
10,000
1000
100
10
1
0.1
0.001
LOD
–3
0
3
6
9
12
14
Days post-infection
C
D
Cynomolgus macaques
AZD7442 0.4 mg/kg
AZD7442 0.04 mg/kg
AZD7442-YTE 40 mg/kg
–2
–1
2
4
5

Figure S3. Human IgG concentrations in serum samples of non-human primates (NHPs) before and after SARS-CoV-2 challenge with antibodies administered in prophylaxis or treatment settings.

Rhesus macaques in prophylaxis groups received an IV infusion of mAb 3 days prior to challenge (40 mg/kg isotype control (n=3), 40 mg/kg AZD7442 (n=3), 4 mg/kg AZD7442 (n=4), and 4 mg/kg AZD7442-YTE (n=4). Rhesus macaques in treatment groups received an IV infusion of 40 mg/kg AZD7442 (n=4) 1 day after challenge. Rhesus macaques were challenged with 10^5 PFU of SARS-CoV-2, split between IT and IN delivery on day 0. Serum was collected on days -3, 0, 1, 2, 4, 7, 10, and 14. Cynomolgus macaques in prophylaxis groups received an IV infusion of mAb 3 days prior to challenge (40 mg/kg isotope control, 4 mg/kg AZD7442, 0.4 mg/kg AZD7442, and 0.04 mg/kg AZD7442; all groups n=3). Cynomolgus macaques in treatment groups received an IV infusion of 40 mg/kg AZD7442(n=3) or 40 mg/kg AZD7442-YTE (n=3) 1 day after challenge. Cynomolgus macaques were challenged with 10^5 $TCID_{50}$ of SARS-CoV-2, split between IT and IN delivery on day 0. Serum was collected on days -3, 0, 1, 2, 4, and 5. (**A**) Geometric mean ± SD human serum IgG concentrations are shown for rhesus macaques that received IV administration of isotype control mAb, AZD7442 or AZD7442-YTE three days prior to virus challenge. One animal in the isotype control mAb group missed being dosed with the isotype control mAb, and as expected, showed no detectable concentrations of circulating human mAb, and was excluded from this analysis of human IgG concentration in serum. **(B)** Geometric mean ± SD human serum IgG concentrations are shown for rhesus macaques that received IV administration of AZD7442 twenty-four hours after virus challenge or isotype control mAb three days prior to virus challenge. One animal in the isotype control mAb group missed

being dosed with the isotype control mAb, and as expected, showed no detectable concentrations of circulating human mAb, and was excluded from this analysis of human IgG concentration in serum. **(C)** Geometric mean ± SD human serum IgG concentrations are shown for cynomolgus macaques that received IV administration of isotype control mAb or AZD7442 three days prior to virus challenge. **(D)** Geometric mean ± SD human serum IgG concentrations are shown for cynomolgus macaques that received IV administration of AZD7442 or AZD7442-YTE twenty-four hours after virus challenge, in comparison to control animals that received isotype control mAb three days prior to virus challenge. Where applicable, arrows indicate day of dosing relative to challenge on day 0. IgG, immunoglobulin G; IN, intranasal; IT, intratracheal; IV, intravenous; LOD, limit of detection; mAb, monoclonal antibody; NHP, non-human primate; SARS-CoV-2, severe acute respiratory syndrome coronavirus 2; PFU, plaque-forming unit; SD, standard deviation; YTE, Immunoglobulin constant heavy chain substitution to extend mAb half-life.

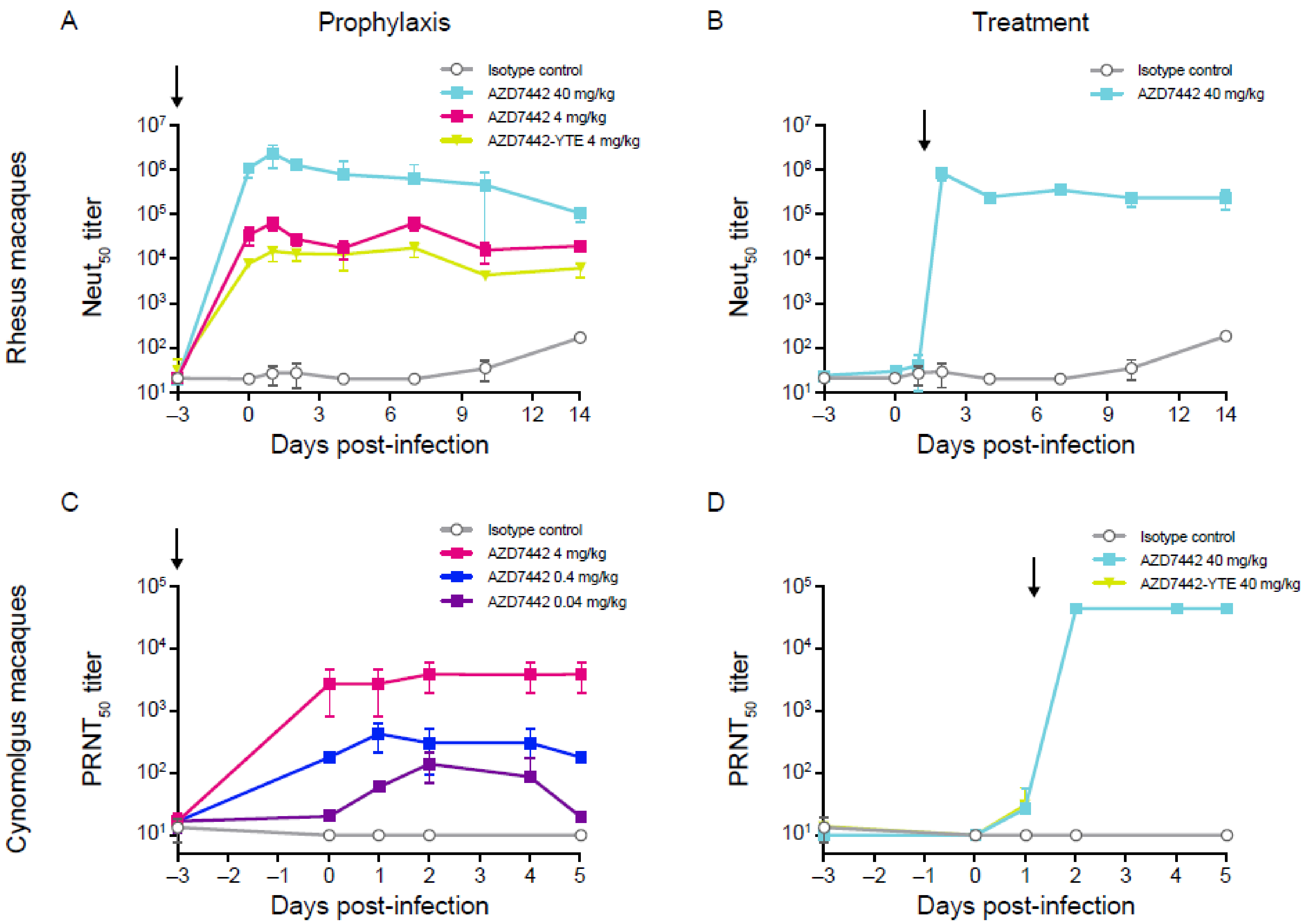
A
Prophylaxis
Isotype control
AZD7442 40 mg/kg
AZD7442 4 mg/kg
AZD7442-YTE 4 mg/kg
Rhesus macaques
Neut50 titer
Days post-infection
B
Treatment
Isotype control
AZD7442 40 mg/kg
Neut50 titer
Days post-infection
C
Isotype control
AZD7442 4 mg/kg
AZD7442 0.4 mg/kg
AZD7442 0.04 mg/kg
Cynomolgus macaques
PRNT50 titer
Days post-infection
D
Isotype control
AZD7442 40 mg/kg
AZD7442-YTE 40 mg/kg
PRNT50 titer
Days post-infection

Figure S4. Serum neutralizing antibody titers in NHPs before or after SARS-CoV-2 challenge infection in prophylaxis or treatment settings.

Rhesus macaques in prophylaxis groups received an IV infusion of mAb at 40 mg/kg isotype control (n=3), 40 mg/kg AZD7442 (n=3), 4 mg/kg AZD7442 (n=4), and 4 mg/kg AZD7442-YTE (n=4) 3 days prior to challenge. Rhesus macaques in the treatment group received an IV infusion of 40 mg/kg AZD7442 (n=4) 1 day after challenge. Rhesus macaques were challenged with 10^5 PFU of SARS-CoV-2, split between IT and IN delivery on day 0. Serum was collected on days -3, 0, 1, 2, 4, 7, 10, and 14. Cynomolgus macaques in prophylaxis groups received an IV infusion of mAb at 40 mg/kg isotope control, 4 mg/kg AZD7442, 0.4 mg/kg AZD7442, and 0.04 mg/kg AZD7442 (all groups n=3) 3 days prior to challenge. Cynomolgus macaques in treatment groups received an IV infusion of 40 mg/kg AZD7442 (n=3) or 40 mg/kg AZD7442-YTE (n=3) 1 day after challenge. Cynomolgus macaques were challenged with 10^5 $TCID_{50}$ of SARS-CoV-2, split between IT and IN delivery on day 0. Serum was collected on days -3, 0, 1, 2, 4, and 5. **(A)** Geometric mean ± SD serum neutralizing antibody ($Neut_{50}$) titers against a SARS-CoV-2 spike protein pseudovirus are shown for rhesus macaques that received IV administration of isotype control mAb, AZD7442 or AZD7442-YTE three days prior to virus challenge. **(B)** Geometric mean ± SD serum neutralizing antibody ($Neut_{50}$) titers against a SARS-CoV-2 spike protein pseudovirus are shown for rhesus macaques that received IV administration of AZD7442 twenty-four hours after virus challenge or isotype control mAb three days prior to virus challenge. **(C)** Geometric mean ± SD serum neutralizing antibody ($PRNT_{50}$) titers against authentic SARS-CoV-2 are shown for cynomolgus macaques that received IV administration of isotype control

mAb or AZD7442 three days prior to virus challenge. **(D)** Geometric mean ± SD serum neutralizing antibody ($PRNT_{50}$) titers against authentic SARS-CoV-2 are shown for cynomolgus macaques that received IV administration of AZD7442 or AZD7442-YTE twenty-four hours after virus challenge, in comparison to control animals that received isotype control mAb three days prior to virus challenge. Where applicable, arrows indicate day of dosing relative to challenge on day 0. IV, intravenous; mAb, monoclonal antibody; NHP, non-human primate; PFU, plaque-forming unit; SARS-CoV-2, severe acute respiratory syndrome coronavirus 2; SD, standard deviation.

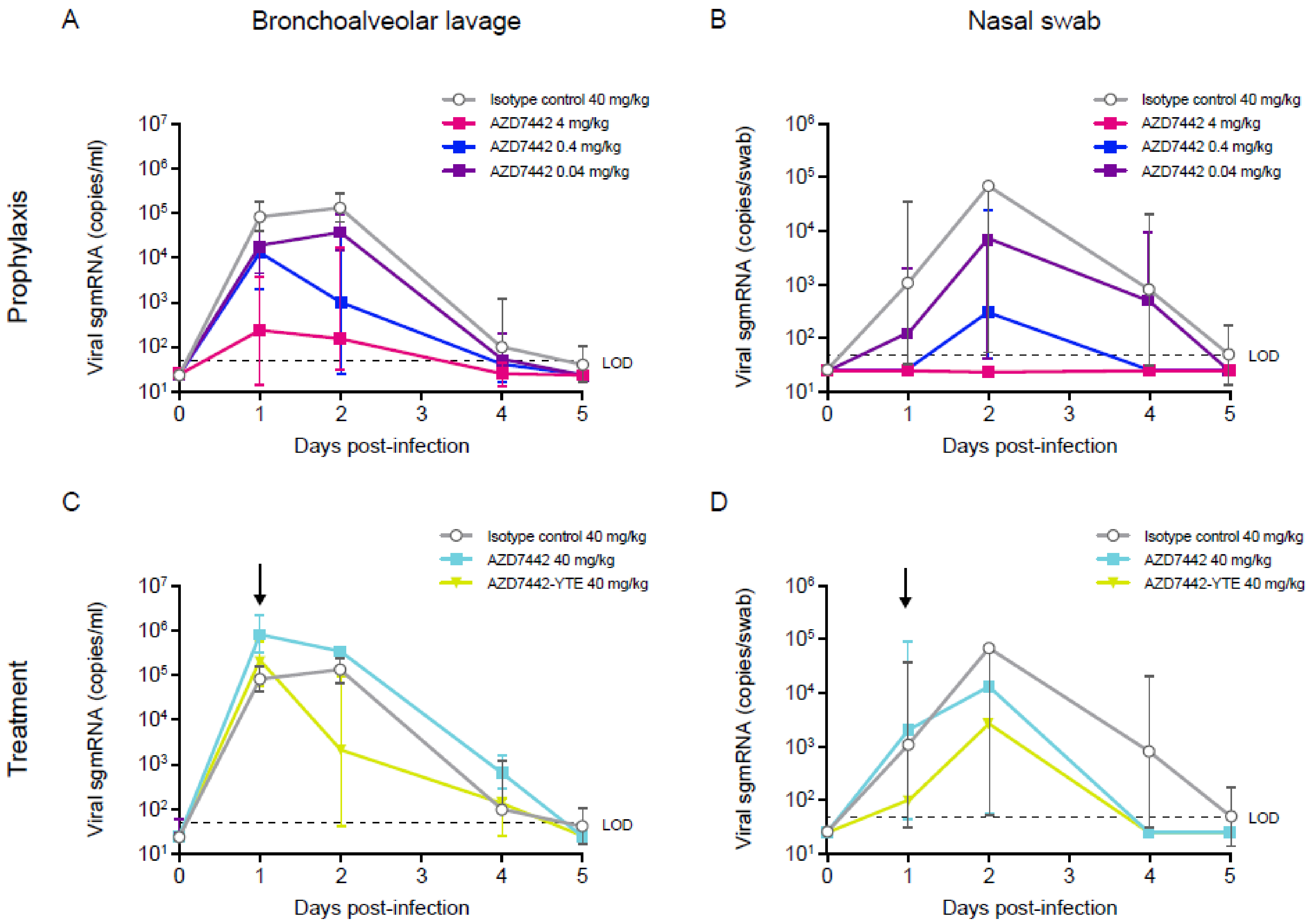
A
Bronchoalveolar lavage
B
Nasal swab
Prophylaxis
Isotype control 40 mg/kg
AZD7442 4 mg/kg
AZD7442 0.4 mg/kg
AZD7442 0.04 mg/kg
Viral sgmRNA (copies/ml)
Viral sgmRNA (copies/swab)
LOD
Days post-infection
C
D
Treatment
Isotype control 40 mg/kg
AZD7442 40 mg/kg
AZD7442-YTE 40 mg/kg
Viral sgmRNA (copies/ml)
Viral sgmRNA (copies/swab)
LOD
Days post-infection

Figure S5. AZD7442 administration protects cynomolgus macaques against SARS-CoV-2 infection in prophylaxis settings.

Cynomolgus macaques in prophylaxis groups received an IV infusion of mAb 3 days prior to challenge (40 mg/kg isotope control, 4 mg/kg AZD7442, 0.4 mg/kg AZD7442, and 0.04 mg/kg AZD7442 (all groups n=3)). Cynomolgus macaques in treatment groups received an IV infusion of 40 mg/kg AZD7442 (n=3) or 40 mg/kg AZD7442-YTE (n=3) 1 day after challenge. Cynomolgus macaques were challenged with 10^5 $TCID_{50}$ of SARS-CoV-2, split between IT and IN delivery on day 0. Serum was collected on days -3, 0, 1, 2, 4, and 5. **(A)** Geometric mean ± SD viral subgenomic RNA (sgmRNA) load is shown for BAL samples from cynomolgus macaques that received IV administration of isotype control mAb or AZD7442 three days prior to virus challenge. **(B)** Geometric mean ± SD viral sgmRNA load is shown for nasal swab samples from cynomolgus macaques that received IV administration of isotype control mAb or AZD7442 three days prior to virus challenge. **(C)** Geometric mean ± SD viral sgmRNA load is shown for BAL samples from cynomolgus macaques that received IV administration of AZD7442 or AZD7442-YTE twenty-four hours after virus challenge, in comparison to control animals that received isotype control mAb three days prior to virus challenge. **(D)** Geometric mean ± SD viral sgmRNA load is shown for nasal swab samples from cynomolgus macaques that received IV administration of AZD7442 or AZD7442-YTE twenty-four hours after virus challenge, in comparison to control animals that received isotype control mAb three days prior to virus challenge. Where applicable, arrows (↓) indicate day of dosing relative to challenge on day

0. BAL, bronchoalveolar lavage; IN, intranasal; IT, intratracheal; IV, intravenous; mAb, monoclonal antibody; sgmRNA, subgenomic messenger RNA; SARS-CoV-2, severe acute respiratory syndrome coronavirus 2; SD, standard deviation.
