# Supplementary material for: The SARS-CoV-2 monoclonal antibody combination, AZD7442, is protective in non-human primates and has an extended half-life in humans
Source: Sci Transl Med. 2022 Jan 25:eabl8124. doi: 10.1126/scitranslmed.abl8124 (PMC8939769; doi:10.1126/scitranslmed.abl8124)
Supplement: Supplementary file 3 — MDAR Reproducibility Checklist [file scitranslmed.abl8124_reproducibility_checklist.docx]

**Materials Design Analysis Reporting (MDAR)**

**Checklist for Authors**

The MDAR framework establishes a minimum set of requirements in transparent reporting applicable to studies in the life sciences (see Statement of Task: doi:10.31222/osf.io/9sm4x.). The MDAR checklist is a tool for authors, editors, and others seeking to adopt the MDAR framework for transparent reporting in manuscripts and other outputs. Please refer to the MDAR Elaboration Document for additional context for the MDAR framework.

**For all that apply, please note where in the manuscript the required information is provided.**

**Materials:**

| **Newly created materials** | **indicate where provided: page no/section/legend)** | **n/a** |
| --- | --- | --- |
| The manuscript includes a dedicated "materials availability statement" providing transparent disclosure about availability of newly created materials including details on how materials can be accessed and describing any restrictions on access. | Main manuscript: *Data and materials availability* |  |
|  |  |  |
| **Antibodies** | **indicate where provided: page no/section/legend)** | **n/a** |
| For commercial reagents, provide supplier name, catalogue number and [RRID](https://scicrunch.org/resources), if available. | - anti-human-Fc-horseradish peroxidase conjugate   - Main manuscript: Materials and Methods, *NHP serum SARS-CoV-2 neutralization assay*   - Supplemetary Materials:     - *SARS-CoV-2 receptor binding inhibition assay*     - *Quantitation of human IgG in NHP serum by ELISA* - Supplementary materials:   - Penta-His antibody: *SARS-CoV-2 receptor binding inhibition assay*   - HRP-conjugated goat anti-human IgG Fc antibody: *SARS-CoV-2 receptor binding inhibition assay*   - Guinea pig complement: *Antibody-dependent complement deposition*   - Fluorescein isothiocyanate (FITC)-conjugated mAb specific for guinea pig C3: *Antibody-dependent complement deposition* |  |
|  |  |  |
| **DNA and RNA sequences** | **indicate where provided: page no/section/legend)** | **n/a** |
| **Short novel DNA or RNA including primers, probes:** Sequences should be included or deposited in a public repository. |  | n/a |
|  |  |  |
| **Cell materials** | **indicate where provided: page no/section/legend** | **n/a** |
| **Cell lines:** Provide species information, strain. Provide accession number in repository **OR** supplier name, catalog number, clone number, **OR** RRID. | FreeStyle 293X or 293F cells   - Supplemetary materials: *Protein expression and purification*   Vero E6 cells   - Main manuscript: Materials and Methods   - *NHP SARS-CoV-2 challenge studies design*   - *Phase 1 serum SARS-CoV-2 neutralization assay* - Supplemetary materials: Materials and Methods, *SARS-CoV-2 neutralization assays* |  |
| **Primary cultures:** Provide species, strain, sex of origin, genetic modification status. |  | n/a |
|  |  |  |
| **Experimental animals** | **indicate where provided: page no/section/legend)** | **n/a** |
| **Laboratory animals or Model organisms:** Provide species, strain, sex, age, genetic modification status. Provide accession number in repository **OR** supplier name, catalog number, clone number, **OR** RRID. | Main manuscript:  - Cynomolgus macaques: Materials and Methods**,** *NHP* *AZD7442 pharmacokinetic study design* and *NHP SARS-CoV-2 challenge studies design* - Rhesus macaques: Materials and Methods, *NHP* *SARS-CoV-2 challenge studies design* |  |
| **Animal observed in or captured from the field:** Provide species, sex, and age where possible. |  | n/a |
|  |  |  |
| **Plants and microbes** | **indicate where provided: page no/section/legend)** | **n/a** |
| **Plants:** provide species and strain, ecotype and cultivar where relevant, unique accession number if available, and source (including location for collected wild specimens). |  | n/a |
| **Microbes:** provide species and strain, unique accession number if available, and source. | SARS-CoV-2 USA-WA1/2020:   - Main manuscript: Materials and Methods, *NHP SARS-CoV-2 challenge studies design* - Supplementary materials, Materials and Methods, *SARS-CoV-2 neutralization assays*   SARS-CoV-2 AUS/VIC01/2020:   - Supplemetary materials, Materials and Methods, *SARS-CoV-2 neutralization assays* - Pseudovirus expressing SARS-CoV-2 spike protein: Supplemetary materials, Materials and Methods, SARS-CoV-2 neutralization assays |  |
|  |  |  |
| **Human research participants** | **indicate where provided: page no/section/legend) or state if these demographics were not collected** | **n/a** |
| If collected and within the bounds of privacy constraints report on age, sex and gender or ethnicity for all study participants. | Main manuscript: Materials and Methods, *Phase 1 clinical study design* |  |

**Design:**

| **Study protocol** | **indicate where provided: page no/section/legend)** | **n/a** |
| --- | --- | --- |
| If study protocol has been pre-registered, provide DOI. For clinical trials, provide the trial registration number **OR** cite DOI. | *Animal studies:* Main manuscript: Materials and Methods   - *NHP SARS-CoV-2 challenge studies design* - *Phase 1 serum SARS-CoV-2 neutralization assay*  *Phase I Clinical Study:* main manuscript, Materials and Methods, *Phase 1 clinical study design and clinical sampling* (NCT number provided) |  |
|  |  |  |
| **Laboratory protocol** | **indicate where provided: page no/section/legend)** | **n/a** |
| Provide DOI **OR** other citation details if detailed step-by-step protocols are available. |  | n/a |
|  |  |  |
| **Experimental study design (statistics details)** | | |
| **For in vivo studies:** State whether and how the following have been done | **indicate where provided: page no/section/legend. If it could have been done, but was not, write not done** | **n/a** |
| Sample size determination | Main manuscript: *Materials and Methods* |  |
| Randomisation | Main manuscript: *Materials and Methods* |  |
| Blinding | Main manuscript: *Materials and Methods* |  |
| Inclusion/exclusion criteria | [Supplementary: *Footnote Figures 4 and S3*] | n/a |
|  |  |  |
| **Sample definition and in-laboratory replication** | **indicate where provided: page no/section/legend** | **n/a** |
| State number of times the experiment was replicated in laboratory. | *Supplementary methods (following assays):* *Antibody binding assay**SARS-CoV-2 receptor binding inhibition assay**mAb binding to human neonatal Fc receptor (FcRn), Fc-gamma receptor (FcγR), and complement C1q**ADCP with neutrophils* |  |
| Define whether data describe technical or biological replicates. | Main manuscript: *Statistical methods* and footnote to Figure 1 |  |
|  |  |  |
| **Ethics** | **indicate where provided: page no/section/legend** | **n/a** |
| **Studies involving human participants:** State details of authority granting ethics approval (IRB or equivalent committee(s), provide reference number for approval. | Main manuscript: Material and Methods, *Phase I Clinical Study* *clinical study design* |  |
| **Studies involving experimental animals:** State details of authority granting ethics approval (IRB or equivalent committee(s), provide reference number for approval. | IACUC numbers provided in Main Manuscript, Materials and Methods, *NHP AZD7442 pharmacokinetic study design* and *NHP SARS-CoV-2 challenge studies design* |  |
| **Studies involving specimen and field samples:** State if relevant permits obtained, provide details of authority approving study; if none were required, explain why. |  | n/a |
|  |  |  |
| **Dual Use Research of Concern (DURC)** | **indicate where provided: page no/section/legend** | **n/a** |
| If study is subject to dual use research of concern regulations, state the authority granting approval and reference number for the regulatory approval. |  | n/a |

**Analysis:**

| **Attrition** | **indicate where provided: page no/section/legend** | **n/a** |
| --- | --- | --- |
| Describe whether exclusion criteria were preestablished. Report if sample or data points were omitted from analysis. If yes report if this was due to attrition or intentional exclusion and provide justification. | [Supplementary Data file S1] | n/a |
|  |  |  |
| **Statistics** | **indicate where provided: page no/section/legend** | **n/a** |
| Describe statistical tests used and justify choice of tests. | Main manuscript: Material and Methods, *Statistical Methods* |  |
|  |  |  |
| **Data availability** | **indicate where provided: page no/section/legend** | **n/a** |
| For newly created and reused datasets, the manuscript includes a data availability statement that provides details for access or notes restrictions on access. | Main manuscript: *Data and materials availability* |  |
| If newly created datasets are publicly available, provide accession number in repository **OR** DOI **OR** URL and licensing details where available. |  | n/a |
| If reused data is publicly available provide accession number in repository **OR** DOI **OR** URL, **OR** citation. |  | n/a |
|  |  |  |
| **Code availability** | **indicate where provided: page no/section/legend** | **n/a** |
| For all newly generated custom computer code/software/mathematical algorithm or re-used code essential for replicating the main findings of the study, the manuscript includes a data availability statement that provides details for access or notes restrictions. |  | n/a |
| If newly generated code is publicly available, provide accession number in repository, **OR** DOI **OR** URL and licensing details where available. State any restrictions on code availability or accessibility. |  | n/a |
| If reused code is publicly available provide accession number in repository **OR** DOI **OR** URL, **OR** citation. |  | n/a |

**Reporting**

MDAR framework recommends adoption of discipline-specific guidelines, established and endorsed through community initiatives. Journals have their own policy about requiring specific guidelines and recommendations to complement MDAR.

| **Adherence to community standards** | **indicate where provided: page no/section/legend** | **n/a** |
| --- | --- | --- |
| State if relevant guidelines (e.g., ICMJE, MIBBI, ARRIVE) have been followed, and whether a checklist (e.g., CONSORT, PRISMA, ARRIVE) is provided with the manuscript. | The following statements have been included: *“International Committee of Medical Journal Editor (ICMJ) guidelines have been followed in regard to Authorship and contribution.”*“Animal Research: Reporting of In Vivo Experiments (ARRIVE, 2.0) guidelines have been followed in regard to the performance of all animal studies.”ARRIVE 2.0 checklist is provided. |  |
